# EEG-based brain–computer interface with immersive virtual reality for phantom limb pain: a single-center pilot neurofeedback trial

**DOI:** 10.3389/fnhum.2026.1697837

**Published:** 2026-04-07

**Authors:** Vincent Roualdes, Saïd Moussaoui, Jean-Marie Normand, Emmanuelle Kuhn, Julien Nizard, Aurélien Van Langhenhove

**Affiliations:** 1 CHU Nantes, Nantes Université, Nantes, France; 2 Nantes Université, École Centrale Nantes, CNRS, LS2N, UMR 6004, Nantes, France; 3 CHU Nantes, Oniris, INSERM, Regenerative Medicine and Skeleton, RMeS, UMR, Nantes Université, Nantes, France

**Keywords:** brachial plexus injury, brain–computer interface, electroencephalography, motor imagery, neurofeedback, phantom limb pain, virtual reality

## Abstract

**Background:**

Phantom limb pain (PLP) is a challenging neuropathic pain condition following limb amputation or brachial plexus injury. Non-pharmacological interventions such as motor imagery training, phantom motor execution and mirror therapy have shown potential to alleviate PLP by engaging sensorimotor circuits, but their effects are debated. We developed GHOST, a portable EEG-based brain–computer interface (BCI) coupled with immersive virtual reality (VR), allowing patients to control a virtual limb via motor imagery in real time, as a neurofeedback-based rehabilitation tool.

**Methods:**

We conducted a single-center exploratory pilot trial to assess the feasibility and preliminary efficacy of this device. Seven patients with chronic upper-limb PLP (amputees or brachial plexus avulsion, pain ≥3/10) underwent 10 training sessions over 2 weeks. Daily pain diaries (distinguishing continuous pain vs. paroxysmal pain episodes) were recorded for 1 month before and 1 month after the intervention, with follow-up to 6 months. Motor imagery ability, anxiety-depression (HADS), and quality of life (SF-36) were also evaluated.

**Results:**

Six patients completed ≥8 sessions. All participants achieved BCI control of the virtual hand, with high success rates (>70%) even as task difficulty increased, demonstrating system feasibility. No adverse events occurred. Compared to baseline, patients experienced a significant short-term reduction in paroxysmal pain (frequency and intensity of pain “flare-ups”), with up to >80% median decrease in weekly cumulated pain episode intensity (*p* < 0.001). Three of five patients also reported around 30% improvement in average daily pain during the first post-training month. HADS anxiety/depression scores showed a non-significant improving trend. By 3–6 months post-training, pain levels had largely returned to pre-intervention values.

**Conclusion:**

This pilot study supports the safety and feasibility of EEG-BCI with VR for PLP and suggests that it can yield short-term analgesic effects, particularly on paroxysmal pain. These findings support the hypothesis that sensorimotor re-engagement could effectively target maladaptive neural processes underlying PLP, while warranting confirmation in controlled trials. Future work will optimize the training protocol and investigate neuroplastic changes associated with this BCI-VR intervention.

## 1 Introduction

Amputation or partial/complete deafferentation of a limb often results in the experience of the “phantom” limb (Kooijman et al., 2000), whereby patients perceive the absent limb through sensory or kinesthetic hallucinations (so-called “phantom sensations”). Frequently, these patients also suffer from a specific type of chronic neuropathic pain—phantom limb pain—that is complex to treat and generally resistant to combined medical and surgical approaches (Ephraim et al., 2005). Two types of pain are described: continuous pain of variable intensity, commonly described as a burning background pain and paroxysmal pain episodes characterized by electric shock-like sensations lasting from a few seconds to several hours, referred to as painful exacerbations. These two pain types can occur independently. The pathophysiology of this pain remains poorly understood. One of the leading proposed mechanisms is “maladaptive plasticity” resulting from an abnormal reorganization of the primary sensorimotor cortex contralateral to the affected limb (Flor et al., 2006). This reorganization is thought to occur as a consequence of non-use or underuse of the sensorimotor brain region following severe deafferentation of the limb. In upper limb cases, the extent of this cortical plasticity appears to correlate with both the incidence and severity of phantom pain (Karl et al., 2001).

Despite a low level of evidence, it has been shown that motor imagery training—where patients imagine movement of the affected limb—can reduce pain, potentially by inducing cortical reorganization (Beaumont et al., 2011). However, as the ability to imagine movement is a subjective cognitive faculty that is difficult to evaluate, its clinical application remains challenging. Other noninvasive therapeutic strategies, such as mirror therapy (Giraux and Sirigu, 2003; Foell et al., 2014), are also frequently used. In mirror therapy, patients observe the movements of the intact limb reflected in a mirror placed symmetrically, thereby creating the visual illusion of the limb’s presence (Ramachandran and Rogers-Ramachandran, 1996) and, in turn, activating the corresponding motor circuit. These considerations render the phenomenon an attractive therapeutic target.

Based on these elements, we developed a noninvasive neurofeedback medical device named GHOST, which combines motor imagery and mirror therapy techniques. Its concept was first presented at the National Congress of the Société Française d’Évaluation et de Traitement de la Douleur (SFETD, Bordeaux, France, 2016). The device falls within the category of brain–computer interfaces (Daly and Wolpaw, 2008) and provides the user with real-time feedback on their imagery of movement in a virtual reality (VR) environment (Leeb and Pérez-Marcos, 2020). Specifically, when a patient—equipped with an electroencephalograph (EEG) connected to a brain decoder—successfully imagines the requested movement (opening/closing of the affected hand), they see, through a VR headset, a first-person view of an avatar performing the corresponding movement. This constitutes active feedback via a motor imagery training program with immersive visual return.

Since its inception, a class III evidence study using a similar principle—albeit substituting magnetoencephalography (MEG) for EEG and a robotic hand for feedback—demonstrated a significant reduction in phantom pain lasting one week, after three days of training, in 12 patients (Yanagisawa et al., 2020b). Although MEG is a powerful diagnostic and research tool, its bulk, complexity, limited availability, and cost make it unsuitable for routine therapeutic deployment. More recently, a multicenter, double-blind randomized controlled trial (Lendaro et al., 2025) evaluated two extended-reality (XR) interventions in 81 patients, comparing phantom motor execution (PME; real-time myoelectric decoding from the residual limb overt execution of phantom movements to control a virtual limb) with phantom motor imagery (PMI; imagined movements). Both groups exhibited substantial reductions in pain, and PME did not demonstrate superiority over PMI (non-significant between-group difference).

Our study provides a proof of concept for a portable device intended for clinical use. The aim was to evaluate both the feasibility and the potential effects of the device on upper-limb phantom pain in patients with either upper limb amputation or severe brachial plexus injury. The intervention is based on the principle of directly targeting maladaptive plasticity within sensorimotor circuits.

## 2 Patients and methods

### 2.1 Patients

Patients were selected from those treated at the Pain Evaluation and Treatment Center of the University Hospital of Nantes (Nantes, France) or referred by specialized centers in the region. They presented with pain fulfilling the characteristics of neuropathic phantom limb pain affecting the upper limb. Inclusion criteria were as follows: an average pain score of at least 3/10 on the Numerical Rating Scale (NRS); a brachial plexus injury or an upper-limb amputation at least at the wrist; partial or complete motor or sensory deficit for more than 6 months; and absence of cognitive disorders or central nervous system lesions, particularly somatosensory ones (verified by brain and spinal MRI in patients with traumatic injuries).

Patients participated in a single-center, pilot, exploratory, uncontrolled, non-randomized, open-label, prospective clinical trial evaluating a noninvasive medical device as a technological innovation. As this was an exploratory study, the sample size (n = 7) was determined based only on the center’s recruitment capacity during the inclusion period, consistent with sample sizes reported in the literature for non-invasive interventions using neurofeedback techniques. The study spanned 7 months according to the schedule, and comprised: (1) a one-month pre-intervention period (m-1) during which subjects recorded daily evaluations of continuous pain (“minimal,” “maximal,” “average” terms refer to patients’ self-reported minimum and maximum pain levels, and their mean pain rating over the course of the day, on the Numerical Rating Scale) and paroxysmal pain (“duration,” “intensity,” “number” of pain peaks); (2) an experimental device usage period consisting of 10 sessions of 2 h each, conducted daily over 2 weeks (w1-w2, 5 sessions per week); and (3) a subsequent follow-up period (w3-w4 and m1-m6). Patients were required to complete at least 8 of the 10 scheduled training sessions over the 2-week period. Daily pain evaluations were recorded by the patients in a self-assessment diary from day 1 to day 30 following the first session, in the same manner as during the pre-intervention period, and then weekly until the end of the inclusion period (m6). Questionnaires assessing anxiety-depression (Hospital Anxiety and Depression Scale, HAD) and quality of life (SF-36) were completed during the information visit, at day 30, and at the end of the protocol. A subjective evaluation questionnaire of motor imagery ability (the French version of the MIQ-RS: Motor Imagery Questionnaire-Revised Second Version) was administered during the information visit.

**Figure 1:**
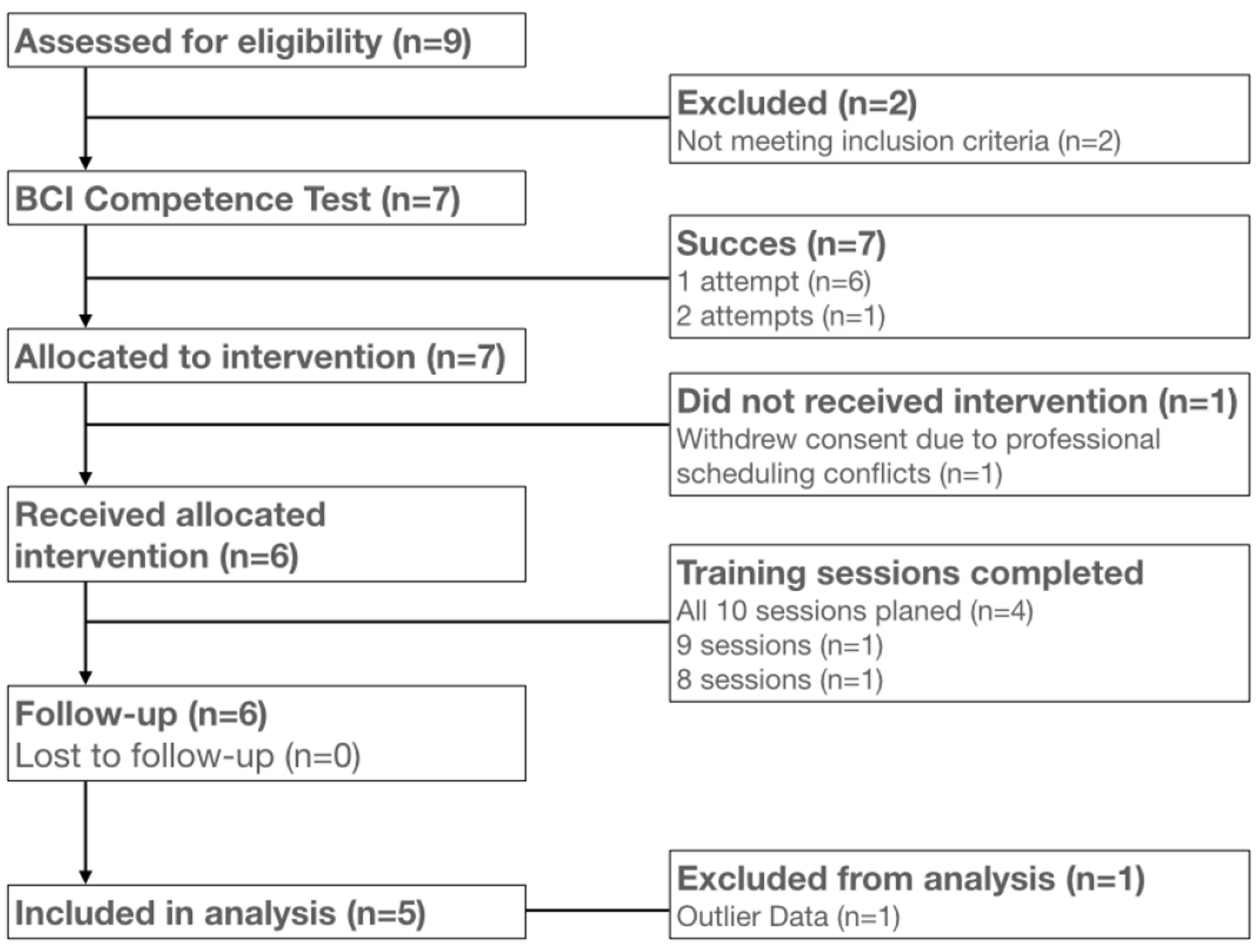
Consolidated standards of reporting trials (CONSORT) flow diagram for the GHOST pilot trial.

A “BCI Competence Test” was performed at day −30. The purpose of this test was to determine whether the EEG-based brain decoder algorithm could detect a specific electrical signature associated with the change between resting state and when the patient executed a series of motor intention tasks with the affected upper limb (see Experimental Procedure). This criterion was mandatory for a patient’s inclusion in the study, as it constituted the indispensable basis for device training. In other words, in the absence of an identifiable signature, no feedback could be triggered. A maximum of three attempts were allowed (Figure 1).

The study was conducted in accordance with the Declaration of Helsinki, approved by the Comité d’Éthique Ouest VI – Brest (France) (N°1,111/DM2), and registered in the EudraCT (n° 2017-A03484-49) and Clinical Trial (n° NCT03889353) registries. Written informed consent was obtained from all patients after detailed explanation of the procedure, the training sessions, and any potential risks.

### 2.2 Presentation of the medical device

**Figure 2:**
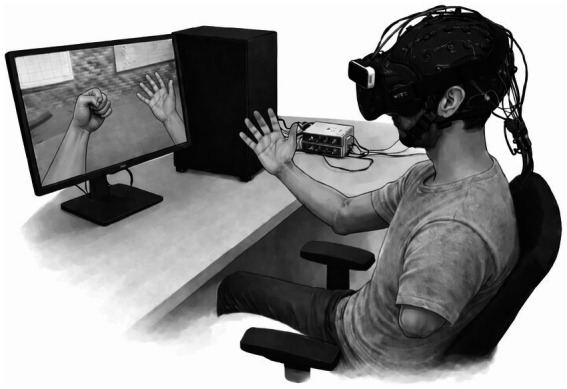
Schematic illustration (artist’s rendering) of the GHOST device during use in a patient with a left upper-limb amputation. The system comprises the following hardware: an HTC vive virtual reality headset; a leap motion controller; a personal computer; a 64-channel EEG amplifier (g.tec Medical Engineering, g.HIamp 80); an elastic EEG cap equipped with 64 active electrodes (g.tec: 64 g.SCARABEO electrodes, one g.SCARABEOgnd ground electrode, and one g.GAMMA ear-clip reference electrode); an active adapter cable (g.HEADbox); and a preamplifier. In addition, two in-house software modules—a signal-processing pipeline (“brain decoder”) and a virtual reality application developed using the Unity 3D engine (v5.6.0f3, 64-bit)—provide visual feedback via a first-person virtual avatar. The patient is seated comfortably. Movements of the intact right upper limb are tracked by the Leap Motion sensor mounted on the VR system and are faithfully reproduced by the avatar displayed to the patient. In the virtual environment, the patient’s amputated limb is replaced by an intact virtual arm embodied by the avatar. This virtual limb is then animated (hand opening and closing movements) when kinesthetic motor imagery of the affected limb is detected from the EEG signal by the pre-calibrated brain decoder developed by our team. The patient performs different exercises along sessions, providing active feedback through a motor imagery training program with immersive visual feedback.

The medical device used was designed and assembled by the University Hospital of Nantes and the École Centrale de Nantes. It belongs to the category of neurofeedback brain–computer interfaces whose general principle is to measure the user’s brain activity in real time, extract qualitative or quantitative information, and provide feedback so that the user can voluntarily adapt their brain activity or mental task. Operating in a closed-loop system, our device is composed of three main hardware components and two software components: a virtual reality headset (Hardware: Head Mounted Display HTC Vive, hand tracking system Leap Motion Controller. Software: SteamVR v1497390325), a computer (Intel Core i7-7800X, base frequency 3.5 GHz, 16 GB RAM, MSI GEForce GTX 1080 Ti Armor OC with 11 GB video memory, running Windows 10), and a 64-channel EEG amplifier (g.tec Medical Engineering g.HIamp 80 CHANNEL AMPLIFIER, CE-marked European class II, certified by TÜV SÜD Product Service GmbH) along with an elastic EEG cap with standard 10–10 system placements plus 86 intermediate positions (64 active g.tec electrodes: g. SCARABEO, 1 g. SCARABEOgnd as ground electrode, 1 g. GAMMA earclip as reference), an adapter cable (g. HEADbox – active), and a preamplifier. In addition, a signal processing software (“brain decoder”) and a virtual reality software (developed with Unity 3D 5.6.0f3, 64-bit) provide the visual feedback via a virtual avatar (Figure 2).

During use, the patient wears the EEG cap and VR headset through which they view a first-person representation of an avatar. Movements are tracked via an infrared sensor (Leap Motion Controller) and replicated by the avatar, thereby virtually restoring body integrity. The affected limb (amputated or paralyzed) is animated via the interface based on the kinesthetic motor imagery performed by the user. Thus, the device integrates neurofeedback based on motor imagery coupled with the principle of mirror therapy in an immersive virtual environment.

The signal processing software, also referred to as the “brain decoder,” was developed jointly by the University Hospital of Nantes and École Centrale de Nantes. Its primary functions are to: (1) acquire EEG signals directly from the EEG device; (2) display these signals in real time to monitor quality; (3) interpret the EEG signals in real time to determine the user’s mental state and transmit this information to the VR software; and (4) guide the user through various mental tasks while recording EEG signals to build a predictive model.

The virtual avatar feedback software was similarly developed by the same institutions. Its main functions are to: (1) immerse the user in a simple virtual world where the avatar does not exhibit the affected limb; (2) react to the user’s mental state—in that when the user thinks of opening and closing their affected hand, the avatar’s corresponding hand performs that movement; and (3) mirror the user’s movements in real time.

The information systems employed comply with medical cybersecurity standards.

### 2.3 Experimental procedure

Each session was conducted in a dedicated room meeting comfort and safety standards, under the joint supervision of the investigator physician and an associated engineer.

The “BCI Competence Test” realized at day −30 was conducted as follows. The patient, with the EEG cap in place, was comfortably seated in front of a computer screen. They first underwent two “baseline” 1-min rest phases. In the first, the patient was instructed to remain calm and relaxed with eyes open and gaze fixed; in the second, to remain calm with eyes closed and without movement. Then, the patient performed 9 series of tasks, each series consisting of 6 trials per task (i.e., 54 trials per task and 162 trials per session). In each series, the interface randomly cued one of the following tasks: (1) imagine opening and closing the right hand at 0.5 Hz (“right hand”); (2) imagine opening and closing the left hand at 0.5 Hz (“left hand”); (3) remain calm, relaxed, with eyes open and fixed (“relax”). All patients were instructed to perform kinesthetic motor imagery, i.e., mentally rehearsing the movement by focusing on the sensation of executing it, without overt movement. Once the recording was complete, the acquired data were immediately analyzed and the software calculated the success rate of the brain decoder’s classification algorithm, which had to reach a threshold of 70% for the patient to be definitively included. In case of failure, the test could be repeated up to three times. The success rate was defined as the ratio of the number of correct decisions to the total number of decisions made by the brain decoder.

**Figure 3:**
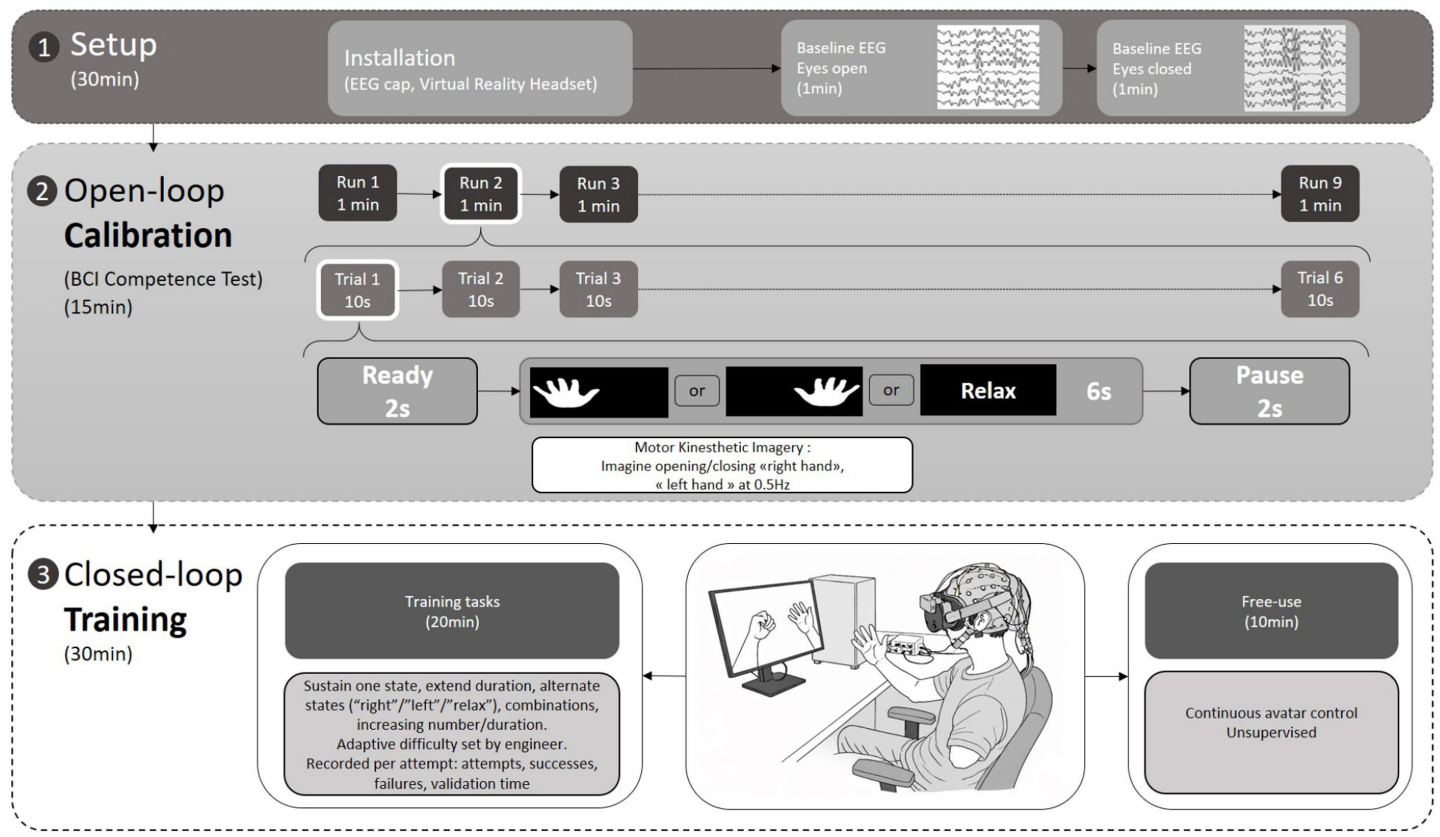
Flowchart of the experimental procedure.

Once enrolled, patients participated in one session per day (excluding weekends) (Figure 3), with a total of 10 device sessions scheduled over 2 consecutive weeks (w1-w2). Each session included the following main steps: (1) Device setup, installation (EEG cap and VR headset) – 30 min; (2) “Open-loop Calibration” phase similar to that in the “BCI Competence Test” – 15 min; (3) “Closed-loop Training” phase followed by “Free Use” – 30 min; and (4) a debriefing period – 30 min.

The “Closed-loop Training” phase, was designed to train the patient to generate increasingly stable and detectable EEG signatures through motor kinesthetic imagery, guided by neurofeedback delivered via the animation of the corresponding virtual limbs of the avatar. To this end, the engineer determined along the session both the quantity and difficulty of the exercises based on the patient’s past performance, fatigue, and emotional state. Adjusting the difficulty aimed to promote progress while preserving motivation. During the initial sessions, patients practiced exercises designed to generate stable EEG signatures (i.e., sustaining each signature for a few seconds). As proficiency increased, the instruction was to extend the duration of signature maintenance. Once mastered, patients were trained to alternate between different mental states according to temporal constraints (For example, one task could be: “right hand” motor imagery for 6 s, “relax” for 3 s, then “left hand” motor imagery for 3 s). Gradually, both the number of alternations and the duration spent in each state were increased whenever feasible. During each training phase, the following parameters were recorded: (1) number of attempts for each task, (2) duration of each task, (3) number of mental states per task, (4) number of successes and failures, as well as (5) the time required to validate an attempt. We did not aim to standardize the training beyond its duration in order to preserve the flexibility required for any individualized rehabilitation process.

The “Free Use” phase, conducted at the end of each session, allowed patients to control the avatar continuously in an unsupervised mode. Although this usage scenario was more natural, it remained cognitively demanding, requiring sustained attention since the brain decoder was active at all times.

### 2.4 Signal processing and “brain decoder”

The brain decoder’s main function is to determine which mental task the patient is currently performing (“right hand” motor imagery, “left hand” motor imagery, or “relax”).

EEG data were acquired either during the BCI “Competence Test” or, at each session, during the “Open-loop Calibration” phase (Figure 3). Signals were first band-pass filtered using a finite impulse response (FIR) filter to retain the frequency band of interest (8–30 Hz), encompassing the alpha (8–12 Hz) and beta (13–30 Hz) rhythms that typically modulate during motor imagery. For each trial, EEG data from 1 to 6 s were extracted and segmented using a 2-s sliding window with 50% overlap, yielding a 3D data structure of size (n_windows × n_channels × n_samples). After filtering and windowing, class-wise statistical characteristics were computed and windows identified as outliers were excluded from the dataset (the artifact-rejection procedure will be described in a future publication).

Following artifact rejection, features were extracted using Common Spatial Patterns (CSP) (Blankertz et al., 2008; Grosse-Wentrup and Buss, 2008)—which computes spatial filters maximizing variance differences between the conditions to be discriminated (MNE-Python implementation) (Gramfort et al., 2013)—and classified using Linear Discriminant Analysis (LDA) [scikit-learn implementation (Pedregosa et al., 2011)]. Decoder performance was assessed using k-fold cross-validation, with accuracy as the performance metric. The number of folds (k) was set to the number of available runs (typically 9 for the “BCI Competence Test” and 6 for the “Open-loop Calibration” phase).

**Figure 4:**
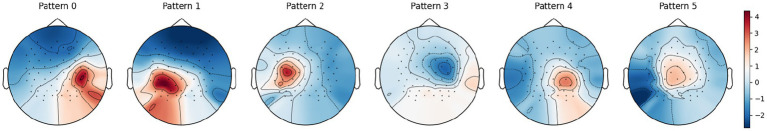
Scalp topographies of EEG spatial patterns (patterns 0–5) derived from data recorded during the BCI competency test (patients 01–05). Each panel shows the scalp distribution of one spatial pattern extracted from the EEG recorded during the BCI competency test, enabling discrimination between the different mental states performed by the patient (“right hand” motor imagery, “left hand” motor imagery, or “relax”). Dots indicate electrode locations. Colors represent the relative contribution (weight) of each sensor to the pattern (arbitrary units; see color bar), with warm and cool colors indicating opposite polarities (the sign is arbitrary). These spatial patterns highlight the cortical regions that contribute most to the decoding of the mental tasks (e.g., sensorimotor areas for motor imagery).

**Figure 5:**
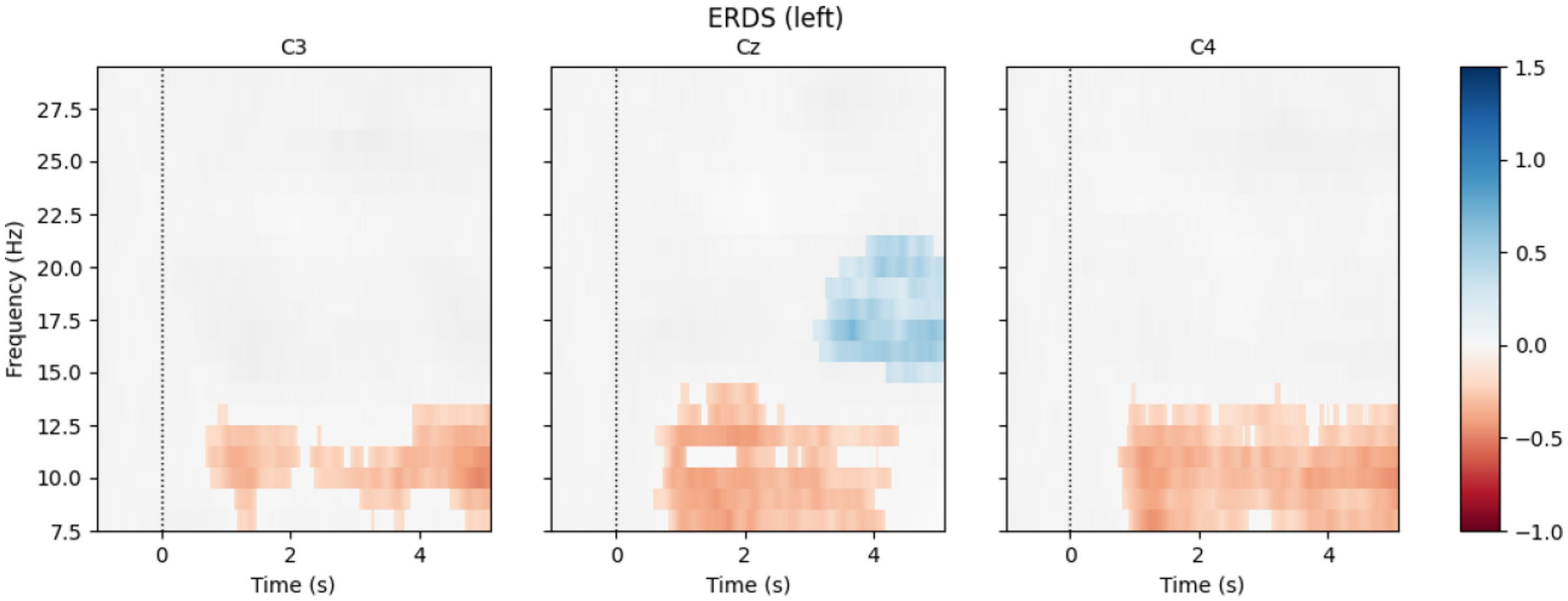
Event-related desynchronization/event-related synchronization (ERD/ERS): time–frequency maps computed at electrodes C3, Cz, and C4 for the motor imagery tasks of subject 01–05, based on data from the BCI Competence Test session. A decrease in signal power in the alpha band is observed during left-hand motor imagery, most prominently at C4, located over the contralateral sensorimotor cortex, as described in literature.

After cross-validation, if the mean CSP–LDA accuracy exceeded 70%, the CSP–LDA model was fitted on the full open-loop dataset and subsequently used during the “Closed-loop Training” phase. An example of CSP-learned spatial patterns is shown in Figure 4. To confirm that the identified signatures reflect genuine sensorimotor EEG activity, ERD/ERS time–frequency spectrograms can be computed. For each open-loop trial, we extracted an EEG segment from −1 s to 6 s. Power spectral density over time was estimated using the Discrete Prolate Spheroidal Sequences (DPSS) multitaper method (implementation available in MNE-Python). ERD/ERS was then expressed as a percent change relative to baseline by subtracting the mean power during the reference interval (−1 to 0 s) and dividing by the mean baseline power. An example computed from an open-loop session of subject 01–05 is shown in Figure 5.

**Figure 6:**
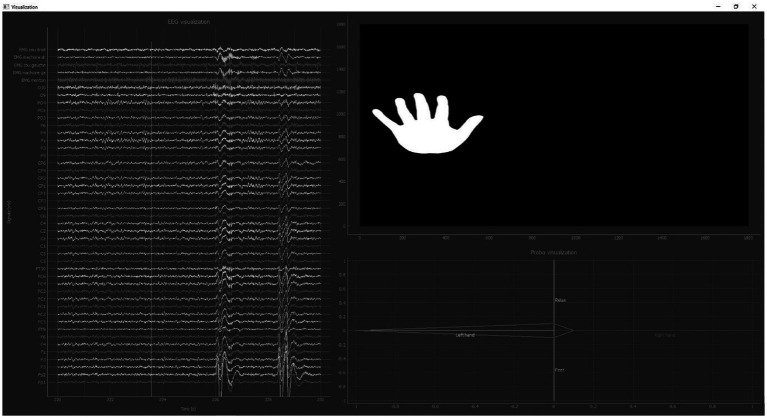
Operator interface during the “Closed-loop training” phase. Left: Raw EEG signal; Right: Top panel showing the instruction provided to the patient during the brain decoder calibration phase (e.g., “left hand” motor imagery) and the bottom panel displaying the real-time classification result (predicted probability of belonging to a given mental state).

During the “Closed-loop Training” phase, EEG was continuously acquired, FIR band-pass filtered, and segmented using a 2-s sliding window with an overlap of 15/16 (corresponding to an update every 62.5 ms, i.e., 16 decisions/s). For each window, the fitted CSP–LDA model was applied to label the signal into one of the three learned classes. The resulting label (Figure 6) was then sent to the VR application to animate the avatar.

### 2.5 Statistical analyses

Given the small sample sizes collected in this study, our analyses primarily relied on descriptive statistics: median, standard deviation, and quartiles.

Given the repeated-measures structure and substantial inter-individual variability in pain trajectories, we employed a linear mixed-effects model (LMM) instead of traditional non-parametric approaches such as the Wilcoxon signed-rank test. LMMs explicitly account for within-subject correlation by incorporating random intercepts for each participant, thereby modeling baseline heterogeneity in pain perception. This approach is particularly suited for longitudinal neurorehabilitation studies with small samples, where inter-subject variability and unbalanced data are expected.

The primary fixed effect of interest was time period, defined in three levels: pre-intervention (m–1), intervention (w1–w2), and post-intervention (w3–w4). Random intercepts were included at the subject level. Model parameters were estimated using restricted maximum likelihood (REML). To assess the global effect of time, we performed a likelihood ratio test (LRT) comparing the full model to a reduced model excluding the period factor. Wald z-tests were used to evaluate the significance of fixed-effect coefficients, with results reported as estimated marginal means (differences between periods), standard errors, and 95% confidence intervals.

All analyses were conducted in Python using the statsmodels library (Seabold and Perktold, 2010), following standard recommendations for mixed-effects modeling of repeated-measures and neurophysiological data (Luke, 2017).

## 3 Results

Seven patients (5 men and 2 women, aged 28–65 years) were consecutively recruited at the University Hospital of Nantes between May 9, 2019, and November 9, 2021, meeting the inclusion criteria (Figure 1). One patient withdrew consent for professional scheduling conflicts. Data from one patient (01–03) could not be analyzed due to the presence of outlier values (see Discussion). Two patients experienced pain related to amputation (one traumatic, the other oncological), while the remaining four had brachial plexus injuries (one radiation-induced complication and three due to traffic accidents causing brachial plexus avulsions). The average duration since the injury was 93 months (range: 7–360 months). All included patients had refractory chronic pain evolving over similar time periods. The main clinical characteristics of the patients participating in the study are detailed in Table 1.

**Table 1:** Demographic and clinical characteristics of the study participants (*n* = 6).

ID	Age/Sex	Diagnosis	Duration (months)	Medical treatments	Surgical treatments	Alternative therapies
01–01	58/F	Radiation-induced right brachial plexus injury (incomplete, C5)	21	Pregabalin 600 mg/day; Paracetamol 3 g/day; Piroxicam 20 mg/day	Brachial plexus neurolysis; C7–T2 DREZotomy	rTMS, self-hypnosis, physiotherapy
01–02	28/M	Traumatic right brachial plexus injury (complete, C5)	44	Duloxetine 90 mg/day; Marinol 7.5 mg/day	Musculocutaneous nerve neurotization via spinal accessory nerve (with intercalated sural nerve graft)	Physiotherapy only
01–03	45/M	Traumatic left arm amputation at shoulder level (work accident)	7	Morphine sulfate 30 mg/day (extended-release); morphine sulfate 40 mg/day (immediate-release); Amitriptyline 20 mg/day; Pregabalin 400 mg/day; Duloxetine 60 mg/day	Left shoulder amputation (work accident)	TENS, mirror therapy, motor imagery training
01–04	65/F	Oncologic left arm amputation at shoulder level (sarcoma)	20	Pregabalin 200 mg/day; Duloxetine 90 mg/day	Left shoulder amputation (sarcoma)	Mirror therapy, motor imagery training
01–05	38/M	Traumatic left brachial plexus injury (C5–C6)	105	Paracetamol 1 g as needed; intolerance to analgesic medications	Neurolysis of C8–T1 roots; nerve graft from C5 to musculocutaneous nerve; 2nd metacarpal elevation and derotation osteotomy; metacarpophalangeal arthrolysis	Qutenza (capsaicin 8% patch), physiotherapy
01–06	56/M	Traumatic right brachial plexus injury (C5–C6)	360	Tramadol/Paracetamol combination (2/day); Pregabalin 250 mg/day; Duloxetine 60 mg/day	C5 nerve graft; spinal accessory nerve neurotization	Mirror therapy, motor imagery training, TENS

For each patient, age, sex, diagnosis, duration of pain since injury (in months), ongoing medical treatments, previous surgical interventions, and alternative or non-pharmacological therapies are reported at inclusion. Diagnoses include traumatic or radiation-induced brachial plexus injuries and upper-limb amputations. Medical treatments reflect stable analgesic regimens at the time of enrollment. Surgical and alternative therapies were performed prior to inclusion and were not modified during the study period.

The reported pain exhibited the typical features of neuropathic phantom pain, with DN4 scores ranging between 5 and 9 [mean 6.83 ± 1.47, median 6.50 (6.00–8.00)]. According to the inclusion criteria, patients had an average pain score of at least 3 on the numerical scale. Three patients experienced between 11 and 20 pain crises per day, with one patient reporting more than 20 crises. Two-thirds of the patients also reported spontaneous pain lasting more than 11 h per day. All patients were receiving multidisciplinary care at pain treatment centers, and the various pharmacological treatments prescribed for neuropathic pain were in line with current recommendations. One patient had undergone a Drezotomy, and four patients with brachial plexus injuries had undergone nerve transfers/grafts/neurolysis. Additionally, five patients had previously tried nonpharmacological treatments (transcutaneous electrical nerve stimulation, self-hypnosis, mirror therapy, motor imagery training, physiotherapy) without long-term efficacy.

### 3.1 Concept of motor imagery training under EEG-BCI control with VR feedback: feasibility

#### 3.1.1 Self-assessed motor imagery ability: MIQ-RS

The MIQ-RS (Motor Imagery Questionnaire-Revised Second Version) evaluates an individual’s ability to imagine motor movements both visually (IMV) and kinesthetically (IMK). The questionnaire comprises seven items where participants are asked to visualize or feel specific movements, rating the ease of performing the imagery on a Likert scale ranging from 1 (very difficult to imagine) to 7 (very easy to imagine).

**Figure 7:**
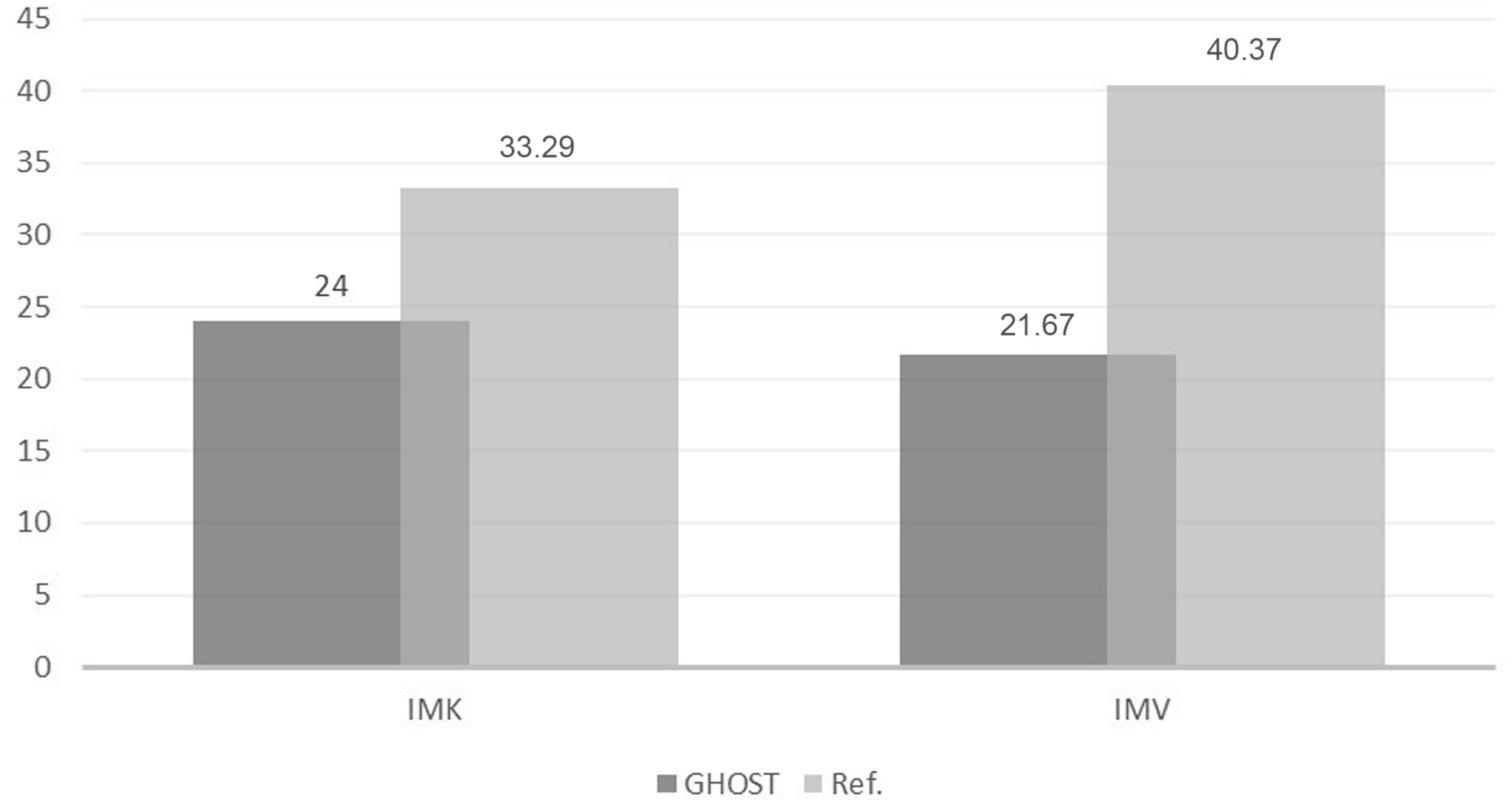
Motor imagery ability scores (MIQ-RS) in GHOST patients. Visual (IMV) and kinesthetic (IMK) motor imagery scores in the GHOST cohort, assessed using the MIQ-RS (7-point scale; higher scores indicate easier imagery; total scores range from 7 to 49 on the ordinate), were lower than normative values reported in healthy subjects in the validation study of the French version of the MIQ-RS, indicating greater difficulty in imagining movements in this patient population.

The mean MIQ-RS scores among patients were 21.67 ± 7.69 [median 25.00 (13.00–27.00)] for visual imagery and 24.00 ± 5.90 [median 23.50 (20.00–26.00)] for kinesthetic imagery (Figure 7). These scores were lower than those observed in healthy subjects in the validation study of the French version of the MIQ-RS (Loison et al., 2013).

Despite these lower scores, all subjects succeeded in generating EEG signatures via motor imagery that were detectable by the “brain decoder” during the “BCI Competence Test.”

#### 3.1.2 Concept of training: feasibility—progressive increase in difficulty

Four patients completed all 10 planned sessions, one completed 9 sessions, and one completed 8 sessions. Each session lasted on average 2 h, including 30 min for setup (cap, headset), 15 min for calibration, and 30 min of training (a fixed duration). The remaining 30–45 min were allocated for reception, installation (dressing/undressing, comfortable positioning), administrative verification, collection of clinical feedback, review of pain diaries, and discussion. The time commitment and cognitive engagement required by the sessions did not constitute a barrier for the patients.

To evaluate the impact of training on avatar control during EEG-BCI feedback sessions, data on the number of sessions, session duration, and exercise difficulty were collected. The exercises involved activating a series of mental tasks corresponding to different states (“relax,” “right hand,” “left hand”). Increasing difficulty was introduced via several parameters (e.g., the maximum time allowed to validate a task, the duration the task is held, and the length of imposed task sequences). A composite score was generated from these elements to provide a clear evaluation of the difficulty presented to the subjects.

**Figure 8:**
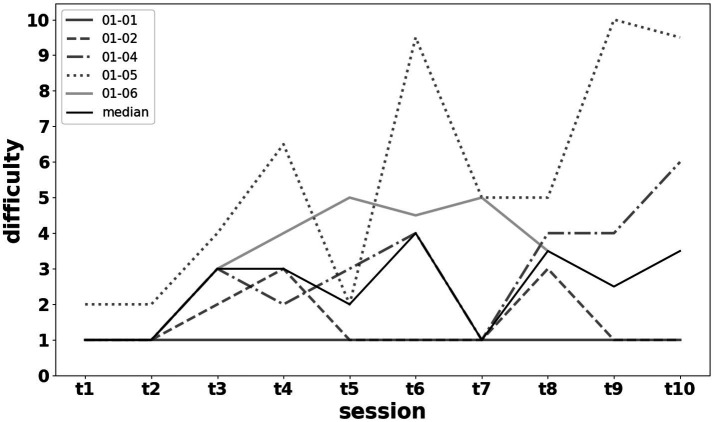
Difficulty of the training exercises for each patient across sessions [t1–t5 performed on days 1–5 during week 1 (w1), and t6–t10 performed on days 8–12 during week 2 (w2)]. The solid black line denotes the median across all participants. During each session, patients performed a sequence of exercises requiring alternation between different mental states under temporal constraints (e.g., right-hand motor imagery for 6 s, relax for 3 s, then left-hand motor imagery for 3 s). When feasible, task difficulty was progressively increased by adding more alternations and/or modifying the duration of each state. The difficulty level was derived from a composite score combining (i) the number of mental states required per task and (ii) completion time expressed as a proportion of the allotted time.

**Figure 9:**
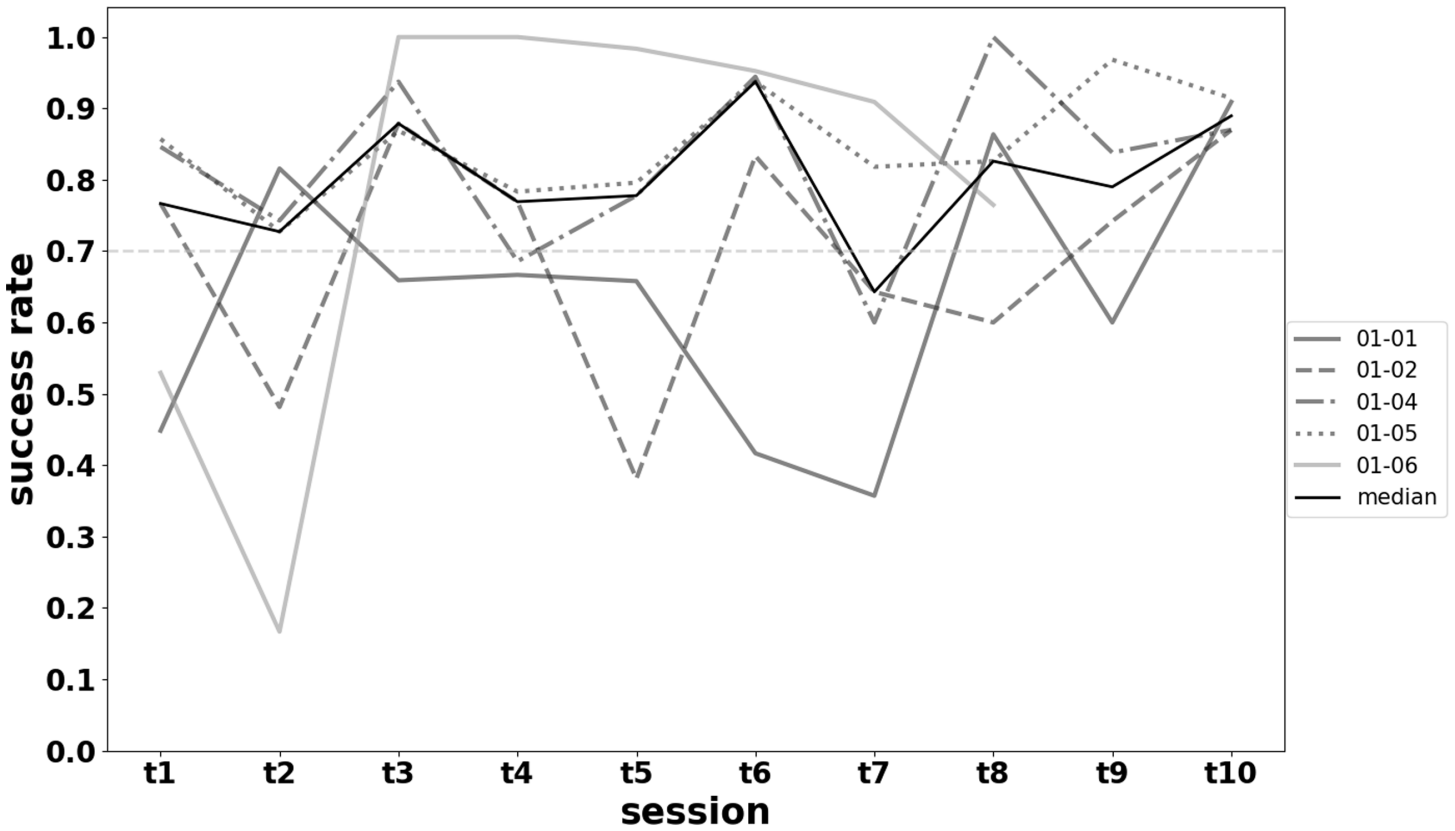
Raw evolution of patients performance during training sessions [t1 to t5 performed on days 1–5 during week 1 (w1), and t6 to t10 performed on days 8–12 during week 2 (w2)]. The success rate was defined as the number of successfully completed exercises divided by the number of attempts within each session, for each patient. The solid black line denotes the median across all participants.

Performance levels remained very high (overall median success rates >70% in exercises) despite an increase in the imposed difficulty level. The results regarding performance and progression in exercise difficulty are shown in Figures 8, 9.

### 3.2 Effect of training on pain and anxiety/depression

#### 3.2.1 Effect on paroxysmal pain

**Figure 10:**
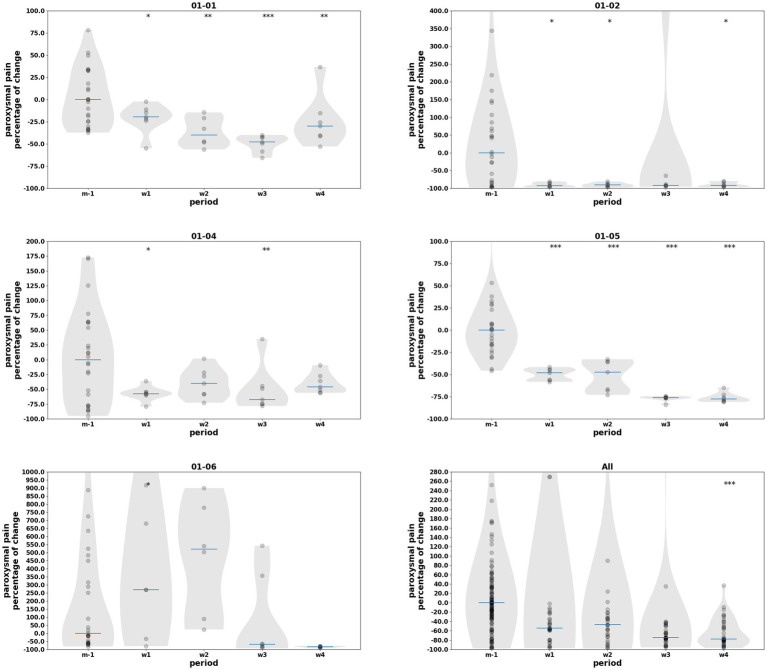
Distribution of relative changes in *paroxysmal pain volume* (defined as the sum of the areas under the curve of individual paroxysmal pain episodes, with each area calculated as pain intensity × self-reported duration of the episode) for each period from baseline (m − 1) to the end of w4, for each patient and for all participants. Violin plots represent the distribution of percentage change relative to baseline (m − 1) for each study period (m − 1, w1, w2, w3, w4). Individual data points correspond to daily measurements pooled across subjects. Horizontal blue bars indicate the median for each period. A progressive reduction in paroxysmal pain is observed during the intervention and follow-up phases, with statistically significant differences across periods [*p* < 0.05 (*), *p* < 0.01 (**), *p* < 0.001 (***)].

Paroxysmal pain showed a pronounced reduction during the intervention period, as reflected by descriptive percentage changes relative to baseline. At the group level, median paroxysmal pain volume decreased by approximately 35% at w1, 55% at w2, 64% at w3, and 86% at w4 compared with the pre-intervention condition (see Table 3). Longitudinal mixed-effects modeling confirmed a significant reduction in paroxysmal pain volume during the post-training phase (w3–w4; coef = −92.6, *p* = 0.001), whereas no significant group-level effect was observed during the early training phase (w1–w2; coef = 6.8, *p* = 0.814) (see Tables 4, 5). Weekly analyses further showed a significant decrease at w4 compared with baseline (coef = −111.4, *p* = 0.001), with earlier weeks showing non-significant or heterogeneous effects at the group level. At the individual level, significant reductions were observed in most patients during both early and late phases, while one patient exhibited a transient early worsening followed by delayed improvement. Long-term follow-up (w12–w23) was not included in statistical analyses, as the change from daily to weekly self-assessments introduced a measurement bias that precluded reliable longitudinal comparison. Individual- and group-level results are illustrated in Figure 10 using violin plots.

#### 3.2.2 Effect on continuous pain

**Figure 11:**
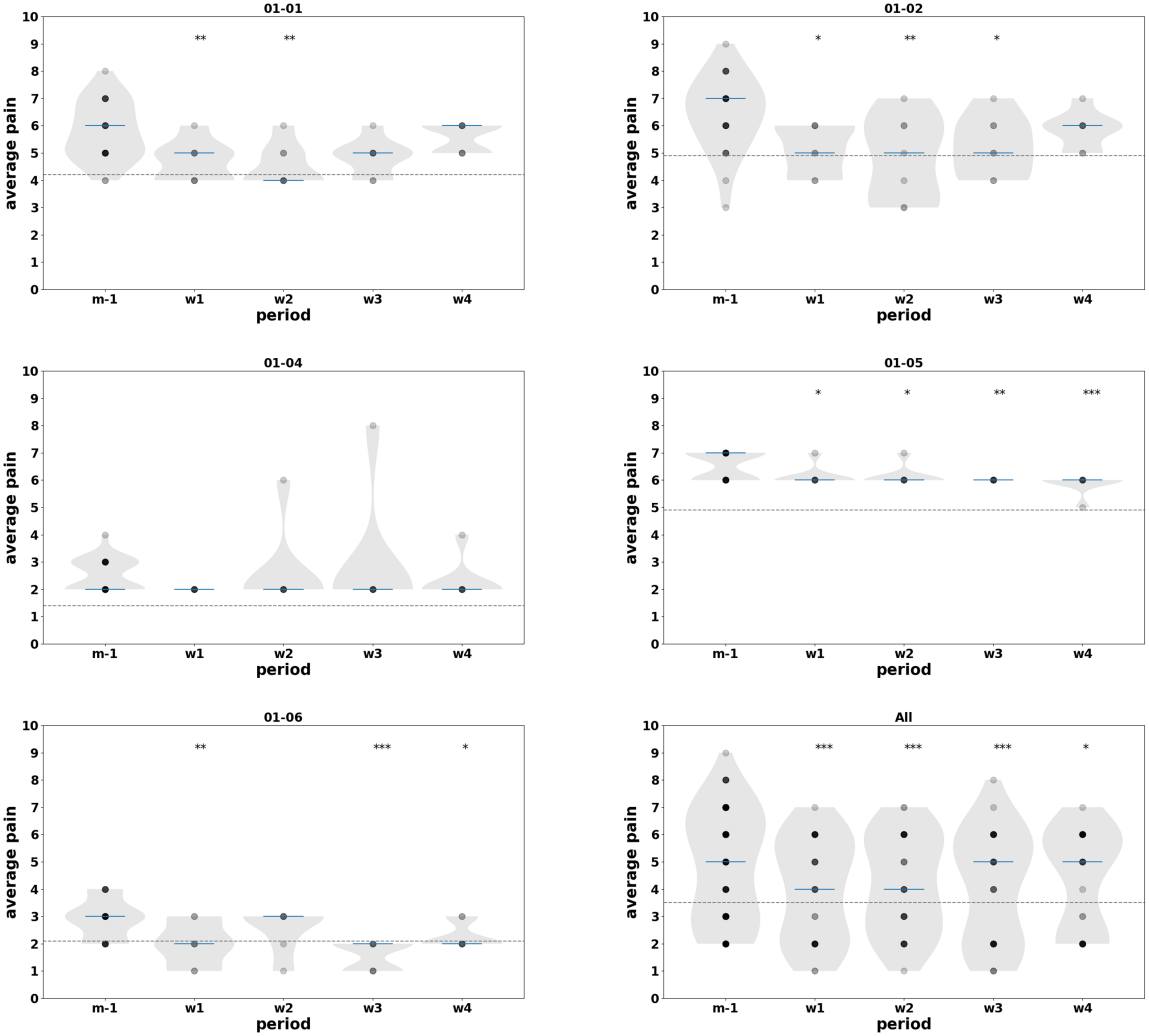
Distribution of continuous pain intensity assessed using the Numeric Rating Scale (NRS) across study periods, from baseline (m − 1) to the end of w4, for each patient and for all participants. Violin plots represent the distribution of absolute NRS scores for each study period (m − 1, w1, w2, w3, w4). Individual data points correspond to daily “average” pain ratings pooled across subjects. Horizontal blue bars indicate the median NRS value for each period. A reduction in continuous pain intensity is observed during the intervention and follow-up phases, with statistically significant differences compared with baseline [*p* < 0.05 (*), *p* < 0.01 (**), *p* < 0.001 (***)].

Average continuous pain intensity (NRS) showed a modest but heterogeneous reduction during the intervention period. At baseline (m − 1), median average pain ranged from low to moderate across patients. Descriptively, most patients exhibited a decrease in median average pain during the intervention weeks, with reductions of up to ~30% in several individuals, while one patient with very low baseline continuous pain (01–04) showed no meaningful change (see Table 2). Linear mixed-effects analyses confirmed these observations, revealing significant group-level reductions in absolute NRS scores compared with baseline across all intervention weeks (w1–w4; coefficients −0.41 to −0.89, *p* ≤ 0.016). Phase-based analyses further demonstrated significant decreases during both the training phase (w1–w2: coef = −0.80, *p* < 0.001) and the post-training phase (w3–w4: coef = −0.61, *p* < 0.001) (see Tables 6, 7). At the individual level, significant improvements were observed in most patients, particularly among those with moderate-to-high baseline continuous pain, whereas changes were smaller and often non-significant when continuous pain was not the predominant pre-intervention symptom. Individual- and group-level results are illustrated in Figure 11 using violin plots.

Long-term follow-up (w12–w23) showed heterogeneous individual trajectories, with some patients maintaining lower average continuous pain compared with baseline, while others returned toward or above pre-intervention levels. At the group level, median continuous pain during this period did not differ from baseline, indicating no sustained long-term effect. Formal statistical analyses beyond day 30 were not performed, as the shift from daily to weekly self-assessments introduced a measurement bias that could not be reliably addressed given the small sample size.

**Table 2:** Descriptive statistics of continuous pain intensity assessed using the Numeric Rating Scale (NRS), reported as minimal, average, and maximal daily pain for each patient and for the whole group across study periods.

		Minimal pain				Average pain				Maximal pain			
Patient	Period	0.25 quantile	Median	0.75 quantile	Variation (% m-1)	Average pain	Median	0.75 quantile	Variation (% m-1)	0.25 quantile	Median	0.75 quantile	Variation (% m-1)
01–01	m-1	2	2	2	NA	5	6	7	NA	8	8	9	NA
01–01	w1	2	2	2	0	4	5	5	−16.66	8	8	9	0
01–01	w2	2	2	2	0	4	4	5	−33.33	7	8	8	0
01–01	w3	2	2	2	0	4,5	5	5	−16.66	7	8	8	0
01–01	w4	2	2	2	0	5	6	6	0	7	8	8.5	0
01–01	w12-w23	2	2	2	0	4.75	5	6.25	−16.66	8	9	9.25	12.5
01–02	m-1	2	3	4	NA	6	7	7	NA	8	8.5	9	NA
01–02	w1	3	3	4	0	4.5	5	6	−28.57	6,5	7	8	−17.64
01–02	w2	2,5	3	4.5	0	3.5	5	6	−28.57	6	7	7,5	−17.64
01–02	w3	3,5	4	4	33.33	4.5	5	6	−28.57	7	7	8	−17.64
01–02	w4	3,5	4	5	33.33	5.5	6	6	−14.28	7	7	7.5	−17.64
01–02	w12-w23	1	1	1	−66.66	4	5	5	−28.57	6	7	7	−17.64
01–04	m-1	0	0	0	NA	2	2	3	NA	4	8	8	NA
01–04	w1	0	0	0	NA	2	2	2	0	6	6	8	−25
01–04	w2	0	0	0	NA	2	2	2	0	6	6	7	−25
01–04	w3	0	0	0	NA	2	2	2	0	6	6	7	−25
01–04	w4	0	0	0	NA	2	2	2	0	6	8	8	0
01–04	w12-w23	0	0	0	NA	4	4.5	5	125	8	8	9	0
01–05	m-1	4	4	5	NA	6	7	7	NA	9	9	9	NA
01–05	w1	4	4	4	0	6	6	6	−14.28	9	9	9	0
01–05	w2	3	3	4	−25	6	6	6	−14.28	8,5	9	9	0
01–05	w3	3	3	3	−25	6	6	6	−14.28	8	8	8	−11.11
01–05	w4	3	3	3	−25	6	6	6	−14.28	7.5	8	8	−11.11
01–05	w12-w23	2	3	3	−25	5	5	6	−28.57	7.5	8	8	−11.11
01–06	m-1	0	0	0	NA	2	3	3	NA	6	7	7.25	NA
01–06	w1	0	1	1	NA	1.5	2	2.5	−33.33	5	6	6.5	−14.28
01–06	w2	0	0	0,75	NA	2.25	3	3	0	6	6.5	7	−7.14
01–06	w3	0	0	0	NA	1	2	2	−33.33	3,5	4	6.5	−42.85
01–06	w4	0	0	0	NA	2	2	2.5	−33.33	5	5	5.5	−28.57
01–06	w12-w23	0	0	1	NA	3	3	3	0	6.75	7	7	0
All	m-1	0	2	4	NA	3	5	7	NA	7	8	9	NA
All	w1	0	2	3,5	0	2	4	6	−20	6	8	9	0
All	w2	0	2	3	0	3	4	6	−20	6	7	8	−12.5
All	w3	0	2	3	0	2	5	6	0	6	7	8	−12.5
All	w4	0	2	3	0	2	5	6	0	6	7	8	−12.5
All	w12-w23	0	1	2	−50	4	5	5	0	7	8	8.75	0

For each pain dimension, values are summarized by the 0.25 quantile, median, and 0.75 quantile. Percentage variation relative to baseline (m − 1) is provided for each period. Baseline values (m − 1) correspond to the pre-intervention period, while subsequent periods include the training phase (w1–w4) and long-term follow-up (w12–w23). Group-level (“All”) values represent pooled distributions across subjects. NA indicates that percentage variation could not be computed (e.g., zero baseline values). This table provides descriptive support for the violin plots and statistical analyses.

**Table 3:** Descriptive statistics of paroxysmal pain volume for each patient and for the whole group across study periods.

Patient	Period	0.25 quantile	Median	0.75 quantile	Variation (% m-1)
01–01	m-1	2.220	2.945	3.900	NA
01–01	w1	2.275	2.370	2.565	−19.52
01–01	w2	1537.5	1770	2242.5	−39.9
01–01	w3	1357.5	1.540	1.695	−47.71
01–01	w4	1745	2065	2.345	−29.88
01–02	m-1	94.562	569.235	1089.747	NA
01–02	w1	30.26	42	82.83	−92.62
01–02	w2	27.745	56.51	71.905	−90.07
01–02	w3	42.5	47	133.25	−91.74
01–02	w4	30.25	46.5	88.5	−91.83
01–04	m-1	153.033	550.133	883.033	NA
01–04	w1	225.166	232.266	250.216	−57.78
01–04	w2	230.091	330.266	414.166	−39.97
01–04	w3	138.15	180.2	293.033	−67.24
01–04	w4	258.166	296.2	376.583	−46.16
01–05	m-1	11.615	14.025	16.71	NA
01–05	w1	6.155	7.27	7.755	−48.16
01–05	w2	4.59	7.36	9,055	−47.52
01–05	w3	3.28	3.36	3.455	−76.04
01–05	w4	2.845	3.12	3.585	−77.75
01–06	m-1	25.5	59	285	NA
01–06	w1	128.5	218	530.5	269.49
01–06	w2	173	367	483	522.03
01–06	w3	11	19	145.5	−67.8
01–06	w4	7	9	11.5	−84.75
All	m-1	22.255	335	1274.015	NA
All	w1	19.56	218	530,5	−34.93
All	w2	20.49	150.3	518	−55.13
All	w3	11	120.2	560.633	−64.12
All	w4	7	46.5	376.583	−86.12

Paroxysmal pain volume was defined as the daily sum of individual paroxysmal pain episodes, with each episode quantified as pain intensity multiplied by its self-reported duration. For each period, values are summarized by the 0.25 quantile, median, and 0.75 quantile. Percentage variation relative to baseline (m − 1) is reported for each subsequent period. Baseline values (m − 1) correspond to the pre-intervention period, while w1–w4 correspond to the intervention and early post-intervention phases. Group-level (“All”) values represent pooled distributions across subjects. NA indicates that percentage variation could not be computed for the baseline condition. This table provides descriptive support for the violin plots and complements the longitudinal mixed-effects analyses.

**Table 4:** Longitudinal comparisons by study phase of the percentage variation in paroxysmal pain score between the baseline condition (m − 1) and the aggregated training (w1–w2) and post-training (w3–w4) phases.

**Patient**	**m-1 VS w1w2**		**m-1 VS w3w4**	
	coef	***p*-value**	coef	***p*-value**
01–01	−30.490	0.001	−38.974	< 0.001
01–02	−130.641	0.002	−91.509	0.032
01–04	−52.742	0.006	−49.700	0.010
01–05	−53.922	< 0.001	−79.758	< 0.001
01–06	319.803	0.011	−201.924	0.099
All	6.810	0.814	−92.645	0.001

Statistical analyses were performed using linear mixed-effects models, with the percentage variation of the paroxysm score relative to m − 1 as the dependent variable. Time period was included as a fixed effect, and subject-specific random intercepts were modeled to account for inter-individual variability. Reported coefficients (coef) correspond to fixed-effect estimates. Negative coefficients indicate a reduction in the paroxysmal pain score compared with baseline (m − 1). *p*-values are reported for each comparison.

**Table 5:** Weekly longitudinal comparisons of the percentage variation in paroxysmal pain score between the baseline condition (m − 1) and each individual study week (w1–w4).

**Patient**	**m-1 VS w1**		**m-1 VS w2**		**m-1 VS w3**		**m-1 VS w4**	
	coef	***p*-value**	coef	***p*-value**	coef	***p*-value**	coef	***p*-value**
01–01	−23.366	0.044	−38.974	0.002	−51.675	< 0.001	−29.448	0.007
01–02	−130.366	0.019	−130.915	0.018	−39.867	0.499	−130.241	0.013
01–04	−61.598	0.013	−43.885	0.076	−69.148	0.009	−35.114	0.135
01–05	−53.285	< 0.001	−54.558	< 0.001	−79.052	< 0.001	−80.288	< 0.001
01–06	357.035	0.025	276.365	0.103	−97.081	0.566	−280.556	0.063
All	17.413	0.637	−4.443	0.906	−67.636	0.085	−111.4	0.001

Linear mixed-effects models were used to assess temporal changes, with time (w1–w4) entered as a fixed effect and subject-specific random intercepts included to account for inter-individual variability. The dependent variable was the percentage variation of the paroxysm score relative to the m − 1 condition. Fixed-effect coefficients (coef) and associated *p*-values are reported. Negative coefficients reflect a decrease in paroxysmal pain score compared with baseline.

**Table 6:** Longitudinal comparisons of “average” continuous pain intensity (NRS) between the baseline condition (m − 1) and the aggregated training (w1–w2) and post-training (w3–w4) phases.

Patient	m-1 VS w1w2		m-1 VS w3w4	
	coef	*p*-value	coef	*p*-value
0101	−1.149	< 0.001	−0.577	0.056
0102	−1.464	< 0.001	−0.893	0.027
0104	−0.234	0.517	0.051	0.887
0105	−0.396	0.005	−0.610	< 0.001
0106	−0.698	0.004	−1.000	< 0.001
All	−0.796	< 0.001	−0.612	< 0.001

Linear mixed-effects models were used, with absolute NRS scores as the dependent variable. Study phase was included as a fixed effect, and subject-specific random intercepts were included to account for inter-individual variability. Fixed-effect coefficients (coef) and associated p-values are reported. Negative coefficients reflect a decrease in continuous pain intensity relative to baseline (m − 1).

**Table 7:** Weekly longitudinal comparisons of average continuous pain intensity assessed using the Numeric Rating Scale (NRS) between the baseline condition (m − 1) and each individual study week (w1–w4).

**Patient**	**m-1 VS w1**		**m-1 VS w2**		**m-1 VS w3**		**m-1 VS w4**	
	coef	***p*-value**	coef	***p*-value**	coef	***p*-value**	coef	***p*-value**
01–01	−1.077	0.006	−1.22	0.002	−0.792	0.057	−0.417	0.262
01–02	−1.321	0.012	−1.607	0.002	−1.131	0.044	−0.714	0.154
01–04	−0.52	0.251	0.051	0.91	−0.52	0.281	0.48	0.265
01–05	−0.396	0.031	−0.396	0.031	−0.538	0.006	−0.663	< 0.001
01–06	−0.929	0.002	−0.429	0.172	−1.429	< 0.001	−0.679	0.015
All	−0.855	< 0.001	−0.737	< 0.001	−0.888	< 0.001	−0.405	0.016

Statistical analyses were performed using linear mixed-effects models, with absolute NRS scores as the dependent variable. Time (w1–w4) was included as a fixed effect, and subject-specific random intercepts were modeled to account for inter-individual variability. Reported coefficients (coef) correspond to fixed-effect estimates and represent changes in absolute NRS scores relative to baseline (m − 1). Negative coefficients indicate a reduction in continuous pain intensity compared with baseline. Associated p-values are reported for each comparison.

**Figure 12:**
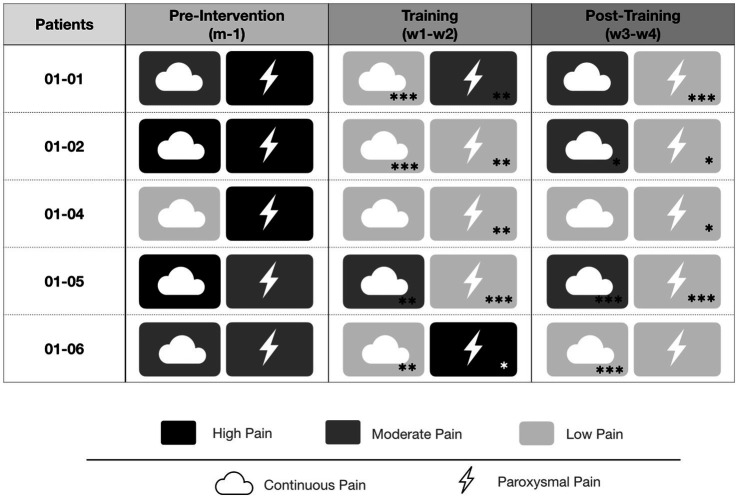
Relative evolution of “average” continuous pain intensity and paroxysmal pain volume for each subject across the three study phases: pre-intervention (m − 1), training (w1–w2), and post-training (w3–w4). Data were derived from daily self-assessments and summarized relative to the baseline median (m − 1). Continuous pain is represented by the cloud icon and paroxysmal pain by the lightning icon. Color coding indicates pain severity (black: high, dark gray: moderate, light gray: low). Asterisks denote statistically significant differences compared with the baseline median m − 1 [*p* < 0.05 (*), *p* < 0.01 (**), *p* < 0.001 (***)]. Absolute values for continuous pain (NRS) and paroxysmal pain volume are provided in the Tables 2 and 3 respectively.

Among the 5 patients for whom usable pain data were available, an improvement in pain was observed during and/or after the training in all cases for paroxysmal pain and in the majority of cases when background pain was the primary complaint (see Figure 12).

#### 3.2.3 Effect on anxiety/depression

Assessments of anxiety/depression and quality of life were collected for all patients using the HAD and SF-36 questionnaires at inclusion (day 1), day 30, and day 180.

Mean HAD scores for depression were 5.00 ± 3.22, 4.33 ± 4.27, and 3.83 ± 4.54, and for anxiety 6.17 ± 3.06, 5.17 ± 2.04, and 5.33 ± 2.88 at Days −30, 30, and 180, respectively. Although there was a nonsignificant reduction in both subscores at the end of the trial, no correlation was found between SF-36 scores and either average pain or paroxysmal pain, likely due to the small sample size.

## 4 Discussion

To better contextualize the development of the device used in this clinical trial, we briefly review the current literature on: (1) the known pathophysiology of phantom limb pain and (2) the wide range of therapeutic strategies available—encompassing surgery, anesthesia, pain management, psychology, rehabilitation, physiotherapy, and occupational therapy—which underscore the complexity of managing these conditions. Specifically, data on mirror therapy, motor imagery, the use of virtual and augmented reality, and Brain–Computer Interface applications in neuropathic and phantom pain will be discussed.

The exact pathophysiology of phantom pain remains unclear, likely involving multifactorial mechanisms including somatic, psychological, and sociological factors similar to other neuropathic pain conditions. Changes have been documented at the peripheral, spinal, and cerebral levels. According to the “central” hypothesis, maladaptive sensorimotor cortical plasticity (Makin and Flor, 2020)—particularly in the primary sensory cortex (S1)—occurs in proportion to pain intensity (Flor et al., 1995). In an attempt to influence this reorganization, therapies targeting the perception of the affected limb (mirror therapy, prosthesis use, motor imagery) have been proposed. Recent neuroimaging and interventional studies, as reviewed by Makin and Flor (2020), however, suggest that the relationship between cortical reorganization, the cortical representation of the intact limb, and phantom pain is not straightforward. Internal and external factors such as prosthetic use, the manner of using the intact limb, disruptions of body schema, and cognitive–affective components (attention, pain memory, control, psychosocial factors, depression, anxiety, emotion, stress response) appear to impact the underlying networks. Bidirectional connections between sensorimotor cortices further influence S1 reorganization and raise questions about the role of subcortical structures involved in motor control (e.g., cerebellum, basal ganglia).

The scientific evidence for the effectiveness of various therapeutic modalities remains weak in view of the substantial literature reporting diverse, pragmatic approaches that combine pharmacological and interventional methods (Erlenwein et al., 2021). Few randomized controlled trials have been published due to recruitment difficulties and the complexity of managing this pathology. No new pharmacological therapy has met the stringent criteria of evidence-based medicine (EBM). Recent literature reviews (Hall and Eldabe, 2018) and a 2016 Cochrane analysis (Alviar et al., 2016), which identified only 14 studies comprising a total of 269 (randomized or quasi-randomized) patients, suggest that treatments with proven albeit limited efficacy include gabapentinoids, opioids, and ketamine. Oral NMDA receptor agonists, botulinum toxin, amitriptyline, and calcitonin do not appear to offer clear benefits. Topical capsaicin may provide transient improvement. By extension, it is considered reasonable to use neuropathic pain agents, opioids, anticonvulsants (gabapentinoids, carbamazepine, oxcarbazepine), antidepressants (duloxetine, venlafaxine), and cannabinoids. The potential cognitive impact of these treatments, which might affect a patient’s capacity to engage with interventional solutions such as that proposed here, is also a concern with respect to cortical plasticity.

Neuromodulation methods have been evaluated, encompassing noninvasive techniques (transcutaneous electrical nerve stimulation tDCS, repetitive transcranial magnetic stimulation rTMS) and invasive techniques typically used for refractory neuropathic pain (peripheral nerve stimulation, spinal cord stimulation, dorsal root ganglion stimulation). Their effects and levels of evidence remain limited and results are mixed (Petersen et al., 2019).

Drezotomy is an invasive, controlled surgical procedure targeting the spinal cord used historically to treat refractory neuropathic pain, including phantom pain. Its effects are predominantly on paroxysmal pain; however, long-term outcomes are contradictory. According to a recent comprehensive review (Mongardi et al., 2021), favorable results were seen in 60.8% of cases in neuropathic pain following brachial plexus injury, but only 35.3% in phantom pain (decreasing to nearly 15% long term), with an average complication rate of 23.6%.

Prosthetic use in amputees appears to reduce phantom pain by decreasing sensorimotor incongruence and restoring body image through the process of embodiment. The analgesic effect is particularly noted with myoelectric prostheses controlled by residual muscle activity (phantom command). Cortical reorganization has been observed following their use (Lotze et al., 1999). Embodiment may be further enhanced by incorporating tactile feedback (Rognini et al., 2019) on the residual limb [e.g., to inform the patient of grip force (Antfolk et al., 2013)]. However, the acceptance rate of these prostheses remains very low (Cordella et al., 2016), and they are not applicable to nonamputee patients with brachial plexus injuries.

Other methods such as mirror therapy (Chan Brenda et al., 2007; Finn et al., 2017; Wang et al., 2021), motor imagery (Moseley, 2006), and virtual or augmented reality (Ortiz-Catalan et al., 2016; Lendaro et al., 2025) have been the subject of systematic reviews (Herrador Colmenero et al., 2018). Twelve studies have been identified, of which nine were of low methodological quality and three of moderate quality. All documented significant reductions in pain (approximately 37.6% reduction on the visual analogue scale [VAS] with 4 weeks of mirror therapy training, 32% reduction on the NRS with augmented reality), albeit without establishing a high level of formal scientific evidence.

Based on these data, recent clinical updates (Erlenwein et al., 2021) advocate for a two-pronged approach: preventive measures at the onset of the disease (reducing postoperative pain, preventing/restoring body schema) and palliative measures for chronic pain (mirror therapy, virtual reality use, myoelectric prostheses, targeted muscle reinnervation surgery). The interventional protocol evaluated in our study aligns with these recommendations. Phantom pain that is refractory appears to predominantly affect the extremities of the upper and lower limbs; thus, interventions are generally focused on the hands and feet.

BCI applications have been very sparsely studied in this indication, and no BCI-based device has yet been commercialized with a dedicated training program. Yanagisawa and colleagues published four articles between 2016 and 2022 (Yanagisawa et al., 2016, 2018, 2020, 2022) describing the effects of neurofeedback training using a BCI based on magnetoencephalography MEG. In total, 14 patients (predominantly with brachial plexus injuries—9 of whom had previously undergone a Drezotomy, and 3 with amputations) were included. They were asked to control a robotic arm, then the image of an upper limb (opening/closing of the hand), and finally a simplified feedback mechanism where a disk’s size varied proportionally to cortical activation elicited by motor intention. The protocols involved training an algorithm to recognize cortical motor signals corresponding to the intention of movement of both the intact and phantom hand, followed by therapeutic training using these decoders. In the first clinical trial, a transient increase in pain was observed when subjects controlled the device using a decoder based on signals from the “phantom” motor cortex, whereas improvement was seen using signals from the intact side. Similar results were found with robotic feedback, 2D visual feedback, and simplified disk feedback. A reduction in pain (measured via VAS) on days 4 and 8 of training (32 and 36%, respectively) was only observed using a “right hand” decoder, whereas the sham decoder yielded no improvement.

However, MEG-based technologies are not compatible with routine clinical use due to their limited availability, high operational cost, and potential contraindications.

**Figure 13:**
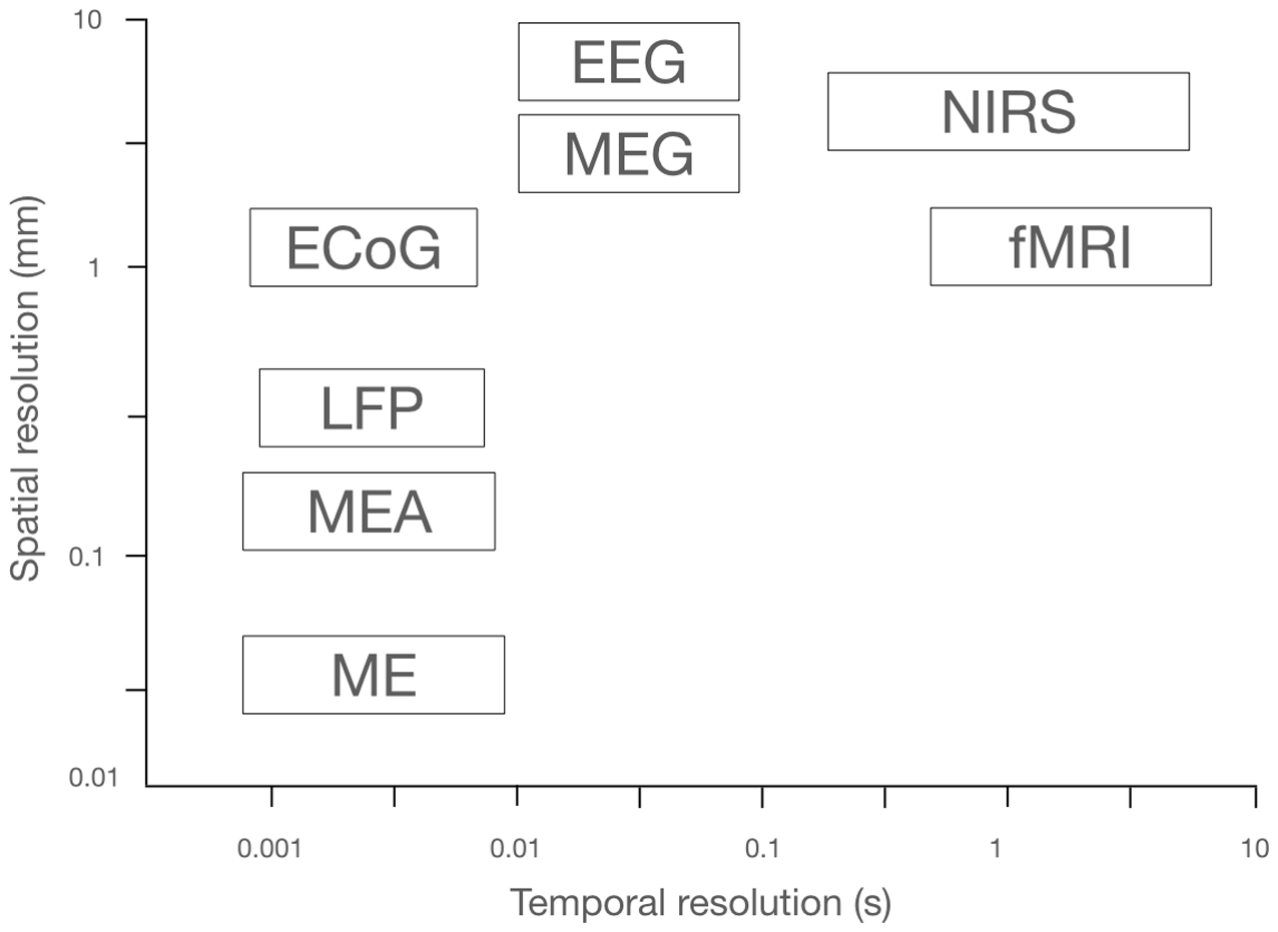
Comparison of the spatial and temporal resolution of various brain activity measurement techniques [adapted from van Gerven et al. (2009)].

A 2021 thesis (Feline Waardenburg – University of Twente – “Mirror Therapy in Virtual Reality by a Brain–Computer Interface for Amputees Experiencing Phantom Limb Pain” https://essay.utwente.nl/87423/1/Waardenburg_BA_EEMCS.pdf) described an experimental device that employed mirror therapy within a virtual reality setting to provide a solution for patients with bilateral impairments (paraplegia, tetraplegia, bilateral amputations). In this pilot project, the task of “pointing to a target with the hand” was controlled via a BCI that did not use EEG as a signal source, but rather functional near-infrared spectroscopy (fNIRS). Although fNIRS offers similar spatial resolution to EEG, its temporal resolution is inferior (Figure 13). This loss of temporal resolution is critical in BCI interactions because the delay between intention and visual feedback is fundamental to embodiment. Tests were performed on 5 healthy subjects in the experimental group and 5 in the control group, but this model has not been evaluated clinically.

The Rubber Hand Illusion (RHI) is an experiment that explores the brain’s plasticity in terms of body perception. In this experiment, a rubber hand is placed in front of the participant while their own hand is hidden. By synchronizing visual stimuli (touches on the rubber hand) with tactile sensations on the hidden real hand, the brain integrates these signals and produces the illusion that the rubber hand is part of the body. This phenomenon illustrates how visual and tactile sensory signals can modify our body schema. Recent studies (Golaszewski et al., 2021; Kanayama et al., 2021) have provided new insights into the neurological mechanisms underlying the RHI and reinforce the hypothesis that visual feedback influences EEG-measured cortical oscillations. These oscillations, associated with visuotactile integration, are known to be altered in phantom pain. The authors demonstrated that virtual reality can induce a stronger RHI than that produced in the real environment, along with significant activations (notably in the gamma band) in cortical processes responsible for visuotactile integration. Additionally, increased theta band activation in frontal regions—linked to error detection and cognitive load—was observed, which correlates with the cognitive demand required to process visuo-spatial incongruence (i.e., when tactile stimulation is congruent or incongruent with visual stimulation). These data further support the use of virtual reality in patients with altered body schema. Moreover, visuotactile incongruence appears to have a lesser impact on subjects in VR compared to real conditions, with equal or superior illusion effects in VR—a factor of paramount importance in BCI applications where delays may compromise temporal congruence of visual feedback.

Our “GHOST” device was conceived before 2016 (concept presented at the 16th SFETD, the French Society for Study and Treatment of Pain) to target maladaptive cortical plasticity in refractory upper-limb phantom pain resulting from brachial plexus injuries or amputation. At that time, the scientific literature pointed to central plasticity as a key mechanism, amenable to noninvasive interventional techniques such as mirror therapy and motor imagery training, despite a generally low level of evidence. Most clinical trials in this domain have been hampered by recruitment challenges due to the epidemiology of these conditions. At that time, the use of virtual or augmented reality and BCI was still emerging, and our understanding of the impact of virtual reality on visuotactile integration was limited. We designed a system to address these issues from multiple angles. Virtual reality offers both immersion and versatility, regardless of the pathology treated (amputation level, complete or incomplete brachial plexus injury, unilateral or bilateral involvement). The choice to use EEG for acquiring brain activity data provides a noninvasive, cost-effective neurofeedback solution. The BCI–VR coupling enables training that combines both mirror therapy and motor imagery practice, while providing the patient with the essential feedback for training and the operator with an objective means of monitoring the session. EEG offers a dynamic and longitudinal evaluation of cerebral activity in a cohort of patients. In essence, “GHOST” was designed to bridge the gap between a novel therapeutic tool and a research instrument.

Although the small sample size limits definitive conclusions, the data collected allowed for an analysis of feasibility, training process with neurofeedback, and analgesic effects. The results are summarized and discussed below.

### 4.1 Effect of training on phantom pain

No meaningful group-level changes were observed in self-reported daily continuous pain intensity (average, minimal, and maximal NRS scores) based on descriptive analyses of the available data. Nevertheless, at the individual level, three patients exhibited a clinically meaningful reduction of approximately 30% in average continuous pain, as illustrated by the shift in median values relative to baseline (m − 1) in the violin plots (dotted reference line Figure 11). These reductions were observed when comparing pre-intervention medians (m-1) with those obtained during the intervention (w1 and w2) and early post-intervention weeks (w3 and w4).

A significant, albeit transient, reduction in paroxysmal pain was observed during the 30 days following the first therapeutic session. Reductions were 35% (w1), 55% (w2), 64% (w3), and 86% (w4) during the first and second intervention weeks, then the first and second post-intervention weeks, respectively. Paroxysmal pain, a particularly disabling component for patients, is seldom evaluated in studies of interventional approaches based on these strategies (Wang et al., 2021). Although we could not establish a correlation between the reduction in paroxysmal pain and quality-of-life parameters in this small series, all patients ultimately reported improvement in at least one component of their pain following treatment (see Figure 12). For example, one patient with relatively low continuous pain showed no significant improvement in average pain but reported a 25% reduction in “maximal” pain (from 8 to 6 on the NRS) alongside a > 65% improvement in paroxysmal pain. Conversely, one patient with high continuous pain did not exhibit significant changes in that measure, but experienced up to 85% improvement in intense paroxysmal pain. Notably, this patient (01–06) was the only one who reported a transient exacerbation of paroxysmal pain during training sessions. To date, dorsal root entry zone lesioning (DREZotomy) is the only other kind of intervention reported to provide meaningful long term relief of paroxysmal deafferentation pain (Chalil et al., 2021; Mongardi et al., 2021); however, it is an invasive strategy with non-negligible morbidity and its efficacy is better established in brachial plexus avulsion–related pain than in post-amputation patients.

The individual summary (see Figure 12) combining continuous (cloud icon) and paroxysmal (lightning icon) pain provides additional insight into these heterogeneous responses. All patients exhibited an improvement in at least one pain dimension (continuous and/or paroxysmal) following the intervention, with the pattern of improvement closely reflecting the predominant pain phenotype observed at baseline. Patients whose pre-intervention pain was primarily characterized by frequent paroxysmal episodes showed the most marked and consistent reductions in paroxysmal pain volume, whereas patients whose main complaint was continuous background pain preferentially exhibited improvements in average NRS scores. In patients presenting with both substantial continuous and paroxysmal pain at baseline, improvements were observed in both components.

Importantly, when a given pain modality was only a minor component of the pre-intervention pain profile, changes in that modality were generally smaller and less likely to reach statistical significance. This pattern suggests a modality-specific effect of the intervention, preferentially targeting the dominant pain component present before treatment, as visually summarized in the synthesis (Figure 12).

Follow-up data collected between days 90 and 180 did not demonstrate a sustained reduction in continuous pain relative to baseline, suggesting a return toward pre-intervention levels in the long term. Formal statistical analysis beyond day 30 was not performed, as the change in the frequency of pain self-assessments—from daily ratings before day 30 to weekly retrospective estimates thereafter—introduced a measurement bias that could not be reliably addressed given the small sample size.

It is noteworthy that the only available data in the literature on BCI-based systems in this indication suggest that feedback controlled by motor intention of the “phantom” limb may transiently increase pain, whereas control using the intact limb’s motor intention reduces pain (Yanagisawa et al., 2020b). These data were not available at the time of our study’s design and may partly explain our findings—indicating that the GHOST device could be used to test this hypothesis. In contrast, Max Ortiz-Catalan’s team published in 2016 (Ortiz-Catalan et al., 2016) a series of 14 upper-limb amputees who underwent 12 sessions using a device that controlled a virtual limb (projected in augmented reality on a 2D screen) via electromyographic sensors on the amputation stump. In that study, significant pain reduction was observed; notably, no pain exacerbation occurred when the phantom network was activated. These findings were confirmed by a multicenter, randomized, double-blind clinical trial conducted by the same team using the same device (Lendaro et al., 2025).

A possible explanation for these discrepancies is the impact of the delay in visual feedback and the level of congruence between the feedback and the motor intention or imagery—both of which are fundamental to embodiment. Myoelectric systems typically exhibit very short response delays and, depending on the sophistication of the processing algorithm, closely approximate the intended movement. In contrast, BCI-based systems have a response delay of one to several seconds, and the response is preprogrammed (e.g., hand opening/closing), thereby being more detached from the intended movement. Nonetheless, myoelectric systems do not replicate the full range of physiological movements and may require counterintuitive training; they are also not accessible to patients with proximal brachial plexus injuries due to denervation.

Mirror therapy, on the other hand, replicates natural movements in real time, albeit only in a mirrored fashion (i.e., when the subject moves their intact limb with the intention of mirroring movements of the phantom limb). In this scenario—similar to Yanagisawa’s BCI strategy—the intact cortex is activated, and the visual feedback is provided on the phantom side via the mirror image. However, mirror therapy does not allow for simultaneous, independent bilateral movements as occurs naturally. It is conceivable that decoupled bimanual activation may be more effective in promoting plasticity due to interhemispheric interaction mechanisms.

BCI systems also enable operation without any actual movement, relying solely on motor imagery—a significant advantage when no myoelectric activity can be captured (e.g., in severe brachial plexus injuries, tetraplegia, paraplegia) or when movement might trigger pain (as in complex regional pain syndrome).

The present findings contribute to an ongoing conceptual debate regarding the mechanisms underlying non-pharmacological, non-invasive interventions for phantom limb pain that rely on interaction with a virtual representation of the missing limb. A growing body of evidence, including the large multicenter randomized controlled trial by Lendaro et al. (2025), suggests that diverse paradigms—ranging from mirror therapy and motor imagery to myoelectric control, BCI-based neurofeedback, VR/XR, and phantom motor execution—can induce substantial and clinically meaningful pain relief. Notably, the absence of superiority of phantom motor execution over motor imagery in the Lendaro et al. trial, despite marked pain reductions in both groups, challenges models positing that overt motor output or stronger peripheral engagement is necessary for analgesia. Instead, these results support the hypothesis that shared higher-level processes, such as the restoration of sensorimotor congruence, embodiment, and coherent action–perception coupling, may play a central role in pain modulation. However, whether these effects arise from common cortical mechanisms, partially distinct but convergent neural pathways, or a combination of specific and nonspecific contextual factors remains unresolved. Taken together, current evidence supports the therapeutic relevance of virtual limb interaction while underscoring the need for mechanistic studies and controlled designs capable of disentangling the relative contributions of motor intention, sensory feedback, embodiment, and neurofeedback to optimize future intervention strategies.

One patient (01–03) exhibited a marked frustration-driven reaction during a failed attempt with the GHOST device at session T6, after which pain-related evaluations abruptly shifted to aberrant values (e.g., a product of daily paroxysm frequency and self-reported duration exceeding 24 h), leading to exclusion of these data from the analysis. This reaction can likely be explained by the patient’s specific context—early post-amputation status following a workplace accident, marked anxiety–depressive symptoms, high expectations, and limited frustration tolerance—and underscores the critical importance of systematically assessing psycho-behavioral and social factors prior to intervention. Such evaluations are mandatory in many countries before neuromodulation procedures such as spinal cord stimulation and may help identify patients who would benefit from psychological support as a first-line approach to prevent adverse emotional reactions. Beyond this individual case, it also highlights the broader challenge of designing non-pharmacological neurotechnological interventions that remain acceptable, tolerable, and sustainable over time. In this regard, the multicenter randomized trial by Lendaro et al. (2025) reported dropout rates exceeding initial projections despite a flexible, patient-centered scheduling strategy, emphasizing that protocol complexity, session duration, cognitive and emotional demands, and frustration tolerance are key determinants of adherence in chronic pain populations. The requirement to complete up to 15 two-hour sessions over several months represents a substantial burden that may limit real-world applicability if not carefully adapted to patient capacity and expectations. Collectively, these observations reinforce the need for careful pre-intervention screening and the development of patient-centered protocols that balance therapeutic intensity with feasibility, as insufficient adaptation may compromise adherence, data interpretability, and ultimately clinical effectiveness.

### 4.2 BCI competence test and “BCI illiteracy”

Approximately 15–30% of users in sensorimotor BCI studies fail to achieve adequate control (Jeunet et al., 2016; Becker et al., 2022)—referred to as “BCI illiteracy.” This may be due either to intrinsic limitations of the brain decoder algorithm and EEG acquisition or to the patient’s difficulty in generating a clear sensorimotor signature due to psychophysiological or cognitive factors (especially following central or peripheral nervous system injuries). For this reason, up to three attempts per patient were planned. All seven patients who underwent the test achieved a classification rate >70% in the binary classification (phantom “hand” vs. “relax”), with only one patient requiring a second attempt. Surprisingly, no patient was excluded from the study during inclusion due to failure of the BCI tests.

Interestingly, better EEG classification results during motor imagery were observed in patients with neuropathic pain due to central nervous system lesions (e.g., in paraplegia), possibly owing to more pronounced signatures compared to the control group (Vuckovic et al., 2015). The absence of BCI illiteracy in our series may be attributable either to chance, given the small sample size, or—as suggested in the literature—to differences in signatures related to the underlying pathology. Further analysis will be conducted to test this hypothesis.

### 4.3 Correlation between self-assessment of motor imagery ability (MIQ-RS) and successful avatar control via motor intention

Motor imagery is a cognitive ability that allows one to mentally simulate movements without triggering actual motor activity. Two primary strategies are recognized: visual imagery (imagining the movement) and kinesthetic imagery (simulating the associated sensations). Motor imagery is used in various rehabilitation programs, with its efficacy seemingly dependent on the patient’s ability to imagine or feel the movement. Pre-assessment of this capability is important, especially given the lack of a reliable clinical measure for an observer to verify performance.

Both imagery strategies activate similar brain regions involved in the actual execution of movement (supplementary motor area, superior and inferior parietal lobules, primary motor cortex, prefrontal regions, inferior frontal gyrus, superior temporal gyrus, primary and secondary sensory cortices, insular cortex, cerebellum, basal ganglia)(Jeannerod, 2001). In many studies, motor imagery—especially when combined with feedback—elicits activations comparable to, or even exceeding, those observed during actual movement (Miller et al., 2010). Numerous studies have demonstrated that coupling motor imagery with conventional rehabilitation enhances outcomes, for instance in the subacute and chronic phases of post-stroke hemiplegia, although the overall level of evidence remains low (López et al., 2019).

To subjectively evaluate the ability of patients in the study to perform motor imagery tasks, the “GHOST” trial utilized the French version of the Movement Imagery Questionnaire – Revised Second Version (MIQ-RS), a test that has undergone scientific validation (Loison et al., 2013). It comprises 7 items per modality, scored from 1 to 7, where lower scores indicate greater difficulty. At the time of study conception, this questionnaire was considered a predictive tool for success in controlling a motor imagery-based BCI (Marchesotti et al., 2016). In conditions such as stroke, brain lesions can impair motor imagery capabilities, hence the importance of assessing this ability prior to initiating this type of rehabilitation. Similarly, we wished to evaluate whether loss of limb use, even if due to a peripheral cause, might affect a patient’s capacity to imagine movement.

The MIQ-RS scores of our patients were markedly lower than the mean scores of healthy subjects (n = 153) from the MIQ-RS validation study (Figure 7), indicating that our patients perceived greater difficulty in performing the motor imagery tasks. Several studies have examined the impact of amputation on patients’ ability to perform motor imagery (MI), but findings have been inconsistent. Lyu et al. reported in amputees (n = 27) reduced performance on an MI task (mental rotation) among participants who experienced vivid phantom limb sensations (Lyu et al., 2016). In our series, the lower MIQ-RS scores did not translate into a higher failure rate in using the BCI device compared to the general population. A publication pointed the absence of a correlation between MIQ-RS results and BCI performance, although it suggested a correlation with manual activity habits and frequency—findings that are difficult to translate to a patient population with deficits (Rimbert et al., 2019).

### 4.4 Virtual reality training with EEG feedback—progressive increase in difficulty

The GHOST protocol is a training program conducted over 15 days with 5 sessions per week. Six out of the 7 enrolled patients were able to complete the sessions; one patient dropped out due to scheduling conflicts. Three of the 6 patients were professionally active during the trial. Although device acceptability was not formally assessed, all 6 patients expressed a desire to continue sessions after the study. Despite the 2-h session duration (with 1 h 15 min of device use, including 30 min of training) and the high concentration demands, no session was interrupted or curtailed.

The training regimen featured a significant, progressive increase in exercise difficulty (Figure 9), tailored to each individual’s performance level and cognitive–emotional state. Difficulty was continuously adjusted based on the experimenter’s pedagogical judgment, without pre-established objective criteria, to facilitate patient progress while avoiding boredom or cognitive overload. This approach allowed patients to maintain a high performance level despite the increasing difficulty (Figure 9). High performance was desirable both to prevent discouragement and to ensure sufficient production of visual feedback to achieve the mirror therapy effect. Variability in performance across sessions was noted and attributed to the inherent fluctuations of any learning process.

It is difficult to ascertain from this study to what extent patients used the feedback to improve their avatar control. This question remains debated in the literature (Horowitz et al., 2021). While neurofeedback-based therapeutic strategies are thought to modulate cortical plasticity, their impact is likely maximized when the patient engages in regular, intensive, and prolonged training under conditions of device mastery. However, as with most published protocols, our study involved subjects who were “naïve” to BCI technology and were in the learning phase rather than achieving full mastery. The analgesic effects observed were transient—a finding consistent with previous studies (Ortiz-Catalan et al., 2016; Finn et al., 2017; Herrador Colmenero et al., 2018; Austin and Siddall, 2021; Austin et al., 2021; Yanagisawa et al., 2022). Given the small sample size, we were unable to reliably determine whether there was a correlation between training progress and improvement in pain. It would therefore be desirable to adopt an initial intensive learning phase followed by a maintenance training program adapted to the kinetics of the therapeutic effect (Yanagisawa et al., 2022).

Other factors—such as engagement, motivation, and cognitive load—must be considered to optimize interventional efficacy. A meta-analysis (Doumas et al., 2021) has highlighted the necessity and superiority of serious game–based rehabilitation programs that could be integrated into training protocols. Although the supervised training duration was 30 min, which patients considered appropriate, no subjective evaluations of cognitive load, motivation, or fatigue were performed. Such assessments would be warranted—especially if the device were to be applied across broader patient populations—to evaluate the impact of sessions and overall acceptability. Some authors (Yanagisawa et al., 2020b) have also evaluated intellectual and verbal quotients, as these functions can affect one’s ability to use technological devices, even when designed for intuitive integration. Furthermore, individuals’ capacity to use feedback to improve performance (Xu et al., 2024; Kim et al., 2025) is highly variable and should be evaluated and taken into account. EEG biomarkers of cognitive load have also been identified (Safari et al., 2024) and could potentially allow real-time adaptation of exercise difficulty without interrupting the patient.

### 4.5 EEG-based differentiation between phantom execution and imagery

In our study, the rehabilitation paradigm relied exclusively on phantom motor imagery (PMI) combined with visual feedback delivered through virtual reality, as opposed to engaging participants in actual phantom motor execution (PME). This methodological choice was grounded in the clinical rationale and empirical evidence available at the time of study design, which supported the efficacy of motor imagery interventions in alleviating phantom limb pain. The neurophysiological distinction between phantom motor execution (PME) and phantom motor imagery (PMI) has been increasingly clarified through EEG-based studies. While both tasks engage motor-related cortical regions and induce event-related desynchronization (ERD) in the sensorimotor *μ* (8–12 Hz) and *β* (13–30 Hz) bands, they differ markedly in spatiotemporal dynamics and signal characteristics. PME, which involves an actual attempt to move the phantom limb, typically produces a transient and more focal ERD localized over the contralateral primary motor cortex (M1), often followed by a clear β-rebound (ERS) after the motor attempt (Zhuang et al., 2020). In contrast, PMI—entirely based on mental simulation—elicits a more sustained but lower amplitude ERD, often distributed over broader fronto-parietal-premotor regions, with reduced post-movement synchronization (Yanagisawa et al., 2020b). Importantly, PME is frequently accompanied by EMG activity in the stump and increased peripheral artifacts, which complicate EEG interpretation and may compromise classifier performance in BCI systems (Zhuang et al., 2020). Conversely, PMI avoids muscular activation and yields cleaner EEG signals, although the neural modulation may be less intense. Additionally, PME has been associated with greater engagement of sensorimotor feedback loops, which may explain its higher classification accuracy in some BCI contexts (Yanagisawa et al., 2022)—but also its potential to exacerbate phantom limb pain in patients with strong maladaptive cortical representations (Yanagisawa et al., 2020b). These findings underscore the functional dissociation between the two conditions and suggest that PMI may offer a safe and technically stable option for BCI applications in amputees—and even more so in cases of brachial plexus injury—particularly when the primary objective is pain relief rather than motor substitution.

### 4.6 Other effects: changes in phantom perceptions

All patients reported qualitative modifications in phantom limb sensations during the sessions. Most notably, paresthesias were commonly experienced within the affected territory during sessions, along with alterations in temperature « perception » or reductions in “pressure” sensations (i.e., “band-like” pain). Patients who previously experienced unpleasant kinesthetic phantom sensations (e.g., a “phantom arm” in a retracted or fixed rotational position) reported transient normalization of the limb’s position (e.g., returning to a neutral posture). Others described a sensation of being able to mentally mobilize the phantom arm, hand, or fingers again, despite having previously experienced a sense of “blockage/paralysis.” These phenomena are classically described among the nonpainful sensory disturbances associated with amputation or deafferentation (Erlenwein et al., 2021).

## 5 Limitations

As a pilot study, several limitations must be acknowledged, including primarily the small sample size, the absence of a control group and randomization, and the heterogeneity of the patient cohort. The simultaneous use of two techniques—mirror therapy like and BCI-VR neurofeedback—precludes determining whether the clinical effect is attributable to either intervention alone or to their combination. The training period was limited to two weeks, and the impact of subsequent maintenance sessions was not evaluated.

It should finally be emphasized that our protocol is a feasibility trial and was not methodologically designed to elucidate the underlying neurophysiological mechanisms of phantom limb pain. Rather, on the basis of previously supported hypotheses (cortical sensorimotor maladaptive plasticity), it aimed to assess their potential clinical relevance. Consequently, it does not allow their formal validation: the pain improvement we observed may also reflect non-specific factors (training effects, cognitive or emotional modulation, etc.). Demonstrating a causal relationship between cortical normalization and pain reduction will therefore require subsequent controlled studies incorporating appropriate assessment modalities (e.g., functional MRI with connectivity analyses, EEG graph signal processing etc.).

## 6 Conclusion

In summary, this pilot study shows that an EEG-based brain–computer interface combined with immersive virtual reality is a feasible and safe intervention for the management of phantom limb pain in amputees and patients with brachial plexus injury. Training patients to control a virtual limb through motor imagery was associated with short-term analgesic effects, particularly a reduction in the severity of paroxysmal pain. The approach is adaptable to different clinical profiles and, owing to the portability of the system, could be implemented across diverse care settings. Nevertheless, given the uncontrolled design and small sample size, these findings should be interpreted cautiously. Future controlled trials are required to confirm efficacy and to disentangle the respective contributions of BCI-mediated neurofeedback and immersive visual feedback. Long-term studies will also be essential to determine how analgesic effects can be sustained, for example through prolonged training or maintenance sessions, and to characterize the underlying neural mechanisms using neuroimaging or electrophysiological markers. More broadly, the present results align with a growing body of evidence suggesting that non-pharmacological, non-invasive approaches involving interaction with a virtual representation of the missing limb—such as mirror therapy, BCI-based interventions, VR/XR, myoelectric interfaces, motor imagery, and phantom motor execution—can provide therapeutic benefit by promoting sensorimotor congruence and embodiment, even though the precise causal mechanisms and optimal therapeutic strategy remain to be established.

## Data Availability

The original contributions presented in the study are included in the article/supplementary material, further inquiries can be directed to the corresponding author.
